# Colorectal cancer with low SLC35A3 is associated with immune infiltrates and poor prognosis

**DOI:** 10.1038/s41598-023-51028-w

**Published:** 2024-01-03

**Authors:** Shuai Lu, Xibo Sun, Huazhen Tang, Jinxuan Yu, Bing Wang, Ruixue Xiao, Jinxiu Qu, Fang Sun, Zhuoya Deng, Cong Li, Penghui Yang, Zhenpeng Yang, Benqiang Rao

**Affiliations:** 1 Department of General Surgery, Beijing Shijitan Hospital, Capital Medical University, Key Laboratory of Cancer Foods for Special Medical Purpose (FSMP) for State Market Regulation, Beijing, 100038 China; 2 Department of Breast Surgery, The Second Affiliated Hospital of Shandong First Medical University, Shandong, 271000 China; 3 Zibo Central Hospital Affiliated to Binzhou Medical College, Zibo, 255020 China; 4 Inner Mongolia Medical University, Hohhot, 010100 China; 5 The Fifth Medical Center of the General Hospital of the People’s Liberation Army of China, Beijing, 100000 China; 6 The First Medical Center of Chinese, PLA General Hospital, Beijing, 100000 China; 7 Department of General Surgery, Qilu Hospital of Shandong University, Jinan, 250012 China

**Keywords:** Cancer, Oncology

## Abstract

The expression level of SLC35A3 is associated with the prognosis of many cancers, but its role in colorectal cancer (CRC) is unclear. The purpose of our study was to elucidate the role of SLC35A3 in CRC. The expression levels of SLC35A3 in CRC were evaluated through tumor immune resource assessment (TIMER), The Cancer Genome Atlas (TCGA), Gene Expression Omnibus (GEO), International Cancer Genome Consortium (ICGC), Human Protein Atlas (HPA), qRT-PCR, and immunohistochemical evaluation. TCGA, GEO, and ICGC databases were used to analyze the diagnostic and prognostic value of SLC35A3 in CRC. A overall survival (OS) model was constructed and validated based on the expression level of SLC35A3 and multivariable analysis results. The cBioPortal tool was used to analyze SLC35A3 mutation in CRC. The UALCAN tool was used to analyze the promoter methylation level of SLC35A3 in colorectal cancer. In addition, the role of SLC35A3 in CRC was determined through GO analysis, KEGG analysis, gene set enrichment analysis (GSEA), immune infiltration analysis, and immune checkpoint correlation analysis. In vitro experiments validated the function of SLC35A3 in colorectal cancer cells. Compared with adjacent normal tissues and colonic epithelial cells, the expression of SLC35A3 was decreased in CRC tissues and CRC cell lines. Low expression of SLC35A3 was associated with N stage, pathological stage, and lymphatic infiltration, and it was unfavorable for OS, disease-specific survival (DSS), recurrence-free survival (RFS), and post-progression survival (PPS). According to the Receiver Operating Characteristic (ROC) analysis, SLC35A3 is a potential important diagnostic biomarker for CRC patients. The nomograph based on the expression level of SLC35A3 showed a better predictive model for OS than single prognostic factors and TNM staging. SLC35A3 has multiple types of mutations in CRC, and its promoter methylation level is significantly decreased. GO and KEGG analysis indicated that SLC35A3 may be involved in transmembrane transport protein activity, cell communication, and interaction with neurotransmitter receptors. GSEA revealed that SLC35A3 may be involved in energy metabolism, DNA repair, and cancer pathways. In addition, SLC35A3 was closely related to immune cell infiltration and immune checkpoint expression. Immunohistochemistry confirmed the positive correlation between SLC35A3 and helper T cell infiltration. In vitro experiments showed that overexpression of SLC35A3 inhibited the proliferation and invasion capability of colorectal cancer cells and promoted apoptosis. The results of this study indicate that decreased expression of SLC35A3 is closely associated with poor prognosis and immune cell infiltration in colorectal cancer, and it can serve as a promising independent prognostic biomarker and potential therapeutic target.

## Introduction

CRC is the third most malignant tumor in the world and the second leading cause of cancer-related death. CRC accounts for 10% of cancer incidence and 9% of cancer mortality^1^. The malignant transformation of CRC involves several steps, and it takes about ten years for a polyp to develop into cancer^2^. The average survival rate of early-stage colorectal cancer patients is 90%, while that of late-stage colorectal cancer patients is only 13.1%^3^. In recent years, screening, diagnosis, and treatment of CRC have made steady progress^4–6^. However, due to the lack of early diagnosis and frequent metastasis and recurrence, the prognosis of CRC patients remains poor, making it a major health problem worldwide. The occurrence of cancer is strongly correlated with abnormal molecular signaling pathways, multi-gene mutations, and epigenetic changes^7^. By combining a large amount of biomedical data, including gene expression profiles, genetic and epigenetic information, with computer technology, it is expected to address this problem^8,9^. Therefore, it is critical to study the detailed molecular mechanisms to identify early screening biomarkers and potential therapeutic targets for CRC.

The study and treatment of tumor metabolism reprogramming has become a hot topic in the last few years^10^. In sufficient oxygen and nutrition conditions, tumor cells still rely on glycolysis for energy, which is known as Warburg effect^11^. Cancer cells multiply quickly under the Warburg effect and resist apoptosis, which contributes to tumor development and occurrence^12^. The solute carrier (SLC) superfamily is considered the second largest gene family in the human genome after the ATP-binding cassette (ABC) transporters, which encode endogenous and exogenous substance transporters^13^. The SLC superfamily not only plays a role in various human diseases but also affects the metabolism and transformation of various drugs, such as chemotherapy drugs and diuretics^14^. SLC35 is a subfamily of nucleotide sugar transporters (NST) of nucleoside sugar transport proteins. The family members encode NST located in the endoplasmic reticulum (ER) and/or Golgi apparatus. These transport proteins transport nucleoside sugars from the cytoplasm to the lumens of these organelles, where most of the sugar conjugates are synthesized^15^. According to studies on small multicellular organisms lacking NST, these transport proteins may contribute to morphogenesis, organogenesis, cell immunity, and tumor metastasis^15^. Since NST is mainly involved in glucose metabolism strongly used by malignant tumors, it is important to understand the SLC35 family for cancer metabolism research^16^.

So far, three proteins of the SLC35A family have been designated as substrates. SLC35A1 is involved in the delivery of 5'-cytidine monophosphate (CMP)-sialic acid, SLC35A2 is involved in the transport of UDP-galactose, and SLC35A3 is involved in the uptake of UDP N-acetylglucosamine. Solute carrier 35A (SLC35A) gene family plays a key role in the occurrence and development of tumors. SLC35A3 is highly expressed in triple-negative breast cancer (TNBC) cell lines HCC1395, HCC1187, and MDAMB436^17^. Studies have shown that SLC35A3 is a pathogenic gene of T-cell lymphoma^18^. SLC35A3 is highly expressed in pancreatic ductal adenocarcinoma and is a prognostic marker for pancreatic ductal adenocarcinoma^19^. However, the exact role of SLC35A3 in the prognosis and biological function of CRC has not been studied.

In this study, we first evaluated the correlation between SLC35A3 expression and clinicopathological features in CRC through multiple public databases, as well as clinical specimens and cell lines. Next, we determined the diagnostic and prognostic value of SLC35A3 in CRC. Based on the expression level of SLC35A3 and multivariable regression results, a prediction model for the overall survival of CRC patients was constructed and validated. We explored the mutation and promoter methylation level of SLC35A3 in colorectal cancer through databases to explore the potential pathogenesis of SLC35A3. Differential genes between the low SLC35A3 group and the high SLC35A3 group were analyzed through gene enrichment analysis to explore the potential biological functions of SLC35A3 in CRC. Considering that the tumor microenvironment plays an important role in tumor development, we also discussed the relationship between the expression of SLC35A3 and immune cell infiltration and immune checkpoints in CRC. In vitro experiments validated that SLC35A3 overexpression inhibit the proliferation and invasion ability of colorectal cancer cells, and promotion apoptosis of colorectal cancer cells. In addition to highlighting the role of SLC35A3 in CRC development, this study also demonstrates its potential as a prognostic biomarker and therapeutic target.

## Materials and methods

### TIMER and HPA tool analysis

The tumor immune estimation resource (TIMER, http://TIMER.cistrome.org) was used to comprehensively analyze the different gene expression levels and tumor-infiltrating immune cells in different types of cancers^20^. We obtained the differential expression of SLC35A3 in tumor tissues and normal tissues in different cancer types through TIMER. In addition, the Human Protein Atlas (http://www.proteinatlas.org/) was used to obtain immunohistochemical data of colorectal cancer tissues and normal tissues.

### RNA sequencing data analysis

Normalized RNA-seq data and corresponding clinical pathology information of 647 colorectal cancer (COAD) and rectal adenocarcinoma (READ) tissues and 51 normal tissues were obtained from The Cancer Genome Atlas (TCGA) database (https://portal.gdc.cancer.gov/). The FPKM data file of 3rd level HTSeq-Fragments was downloaded. The main clinical pathological characteristics of CRC patients are shown in Table 1. Some patients' clinical information was incomplete and were excluded only in the case of relative lack of specific clinical factors. In addition, to validate the expression of SLC35A3 mRNA in CRC patients, we downloaded the original gene maps of GSE21510^21^ and GSE87211^22^ from the Gene Expression Omnibus (GEO, https://www.ncbi. nlm.nih.gov/geo/), and obtained a dataset of colorectal cancer patients from the International Cancer Genome Consortium (ICGC, https://dcc.icgc.org/).

**Table 1:** Correlation study between SLC35A3 expression and clinical pathological parameters in colorectal cancer patients.

Characteristic	Low expression of SLC35A3 (n = 322)	High expression of SLC35A3 (n = 322)	*p*
T stage, n (%)			0.602
T1	8 (2.5%)	12 (3.8%)	
T2	51 (16%)	60 (18.7%)	
T3	224 (70%)	212 (66%)	
T4	37 (11.5%)	37 (11.5%)	
N stage, n (%)			**0.004**
N0	165 (51.8%)	203 (63.3%)	
N1	80 (25%)	73 (22.7%)	
N2	74 (23.2%)	45 (14%)	
M stage, n (%)			0.331
M0	237 (82.6%)	238 (86%)	
M1	50 (17.4%)	39 (14%)	
Pathologic stage, n (%)			**0.041**
Stage I	48 (15.5%)	63 (20.1%)	
Stage II	108 (34.8%)	130 (41.5%)	
Stage III	103 (33.2%)	81 (26%)	
Stage IV	51 (16.5%)	39 (12.4%)	
Age, n (%)			0.937
< = 65	139 (43.2%)	137 (42.5%)	
> 65	183 (56.8%)	185 (57.5%)	
Residual tumor, n (%)			0.568
R0	237 (91.5%)	231 (92%)	
R1	2 (0.8%)	4 (1.6%)	
R2	20 (7.7%)	16 (6.4%)	
CEA level, n (%)			0.789
< = 5	132 (63.8%)	129 (62%)	
> 5	75 (36.2%)	79 (38%)	
Perineural invasion, n (%)			0.776
No	76 (73.1%)	99 (75.6%)	
Yes	28 (26.9%)	32 (24.4%)	
Lymphatic invasion, n (%)			** < 0.001**
No	149 (51.4%)	201 (68.8%)	
Yes	141 (48.6%)	91 (31.2%)	
Gender, n (%)			0.636
Female	147 (45.7%)	154 (47.8%)	
Male	175 (54.3%)	168 (52.2%)	

Analysis was performed using the chi-square test. Statistically significant values are shown in bold (*p* < 0.05).

### Patients and clinical tissue samples

We obtained tumor tissues and corresponding adjacent normal tissues of 82 primary colorectal cancer patients admitted to the Gastrointestinal Surgery Department of Zibo Central Hospital in 2022. All CRC patients underwent curative surgery, and the pathological diagnosis was CRC without other malignant tumors. None of the patients received preoperative radiotherapy, neoadjuvant chemotherapy, or other special treatment. All patients signed a written informed consent, and this study was approved by the Medical Ethics Committee of Zibo Central Hospital. This study was conducted in accordance with the Helsinki Declaration. All methods were performed in accordance with relevant guidelines and regulations.

### Diagnosis and prognosis analysis

Based on the colorectal cancer datasets from TCGA, GEO, and ICGC databases, the ability of SLC35A3 mRNA expression levels to distinguish between colorectal cancer and healthy tissues was evaluated using ROC analysis. Based on the TCGA-COADREAD dataset, the correlation between SLC35A3 expression and the prognosis of CRC patients was analyzed using overall survival (OS), disease-specific survival (DSS), and progression-free interval (PFI). In addition, the GSE28722 dataset in the GEO database and the Kaplan–Meier Plotter tool (https://kmplot.com/analysis/) were used to validate the correlation between SLC35A3 expression and the prognosis of CRC patients.

### Construction and validation of the nomogram based on SLC35A3 expression

We conducted univariable and multivariable Cox regression analysis to evaluate whether SLC35A3 can be used as a protective factor for CRC. The clinical parameters involved include age, sex, T stage, N stage, M stage, pathological stage, presence or absence of colonic polyps, and residual tumors. We included multivariable Cox regression variables, and generated nomographs and calibration charts through rms package [version 6.2–0] and survival package [version 3.2–10] to predict the total survival period of 1, 3 and 5 years^23,24^. The diagonal line is used as the best predictor, and the concordance index (C-index) was used to determine the discriminative ability. The performance of the nomogram plot was validated by ROC curves at 1 year, 3 years, and 5 years using TNM staging as a control.

### Genetic mutation and promoter methylation analysis

We used the cBioPortal tool (https://www.cBioPortal.org/) to obtain the mutation frequency, mutation type, and mutation sites of the SLC35A3 protein structure in CRC. In addition, the three-dimensional structure of SLC35A3 was displayed through cBioPortal. The UALCAN tool (http://ualcan.path.uab.edu/) was used to detect the promoter methylation level of SLC35A3 in CRC and adjacent normal tissues.

### GO analysis and KEGG analysis

The TCGA-CADREAD dataset was used to divide tumor samples into low-level SLC35A3 group and high-level SLC35A3 group. The DESeq2 package [version 1.26.0] of R was used to obtain differentially expressed genes between the low SLC35A3 group and the high SLC35A3 group^25^. Differential genes were screened based on |logFC|> 1 and P.adj < 0.05. We analyzed the differential genes through Gene Ontology (GO)^26^ enrichment in three modules: Biological Process (BP), Molecular Function (MF), and Cellular Component (CC). In addition, Kyoto Encyclopedia of Genes and Genomes (KEGG)^27,28^ analysis was performed on the differential genes. The clusterProfiler software package [version 3.14.3] was used for enrichment analysis of differential genes^29^. P.adjust < 0.05 was considered statistically significant.

### GSEA enrichment analysis

Gene set enrichment analysis (GSEA) can be used to evaluate whether a pre-defined set of genes shows a statistically significant difference between two biological states^30^. Based on the TCGA database data, the tumor samples were divided into low-level and high-level SLC35A3 groups. The clusterProfiler R package (version 3.6.0) was used to perform GSEA analysis on significantly differentially expressed genes between the low SLC35A3 group and the high SLC35A3 group. C2 (C2.all.v7.2.symbols.gmt) was used for GSEA. A false discovery rate (FDR) < 0.025 and p.adjust < 0.05 were considered significantly enriched.

### Immune cell infiltration analysis and immune checkpoint analysis

A previous study has identified a gene marker that labels 24 immune cells^31^. Based on the TCGA-COADREAD dataset and the GSVA R package, the single-sample Gene Set Enrichment Analysis (ssGSEA) method was used to identify the level of immune cell infiltration in tumors^32^. Additionally, based on the TCGA-COADREAD dataset, correlation analysis between SLC35A3 and immune checkpoints in the dataset was performed using R (version 3.6.3), and the analysis results were visualized using the ggplot package.

#### Cell culture and transfection

Human colon epithelial cells NCM460 and CRC cell lines (including HCT116, HT29, and SW620) were purchased from Procell Life Science & Technology Co., Ltd. (Procell, Wuhan, China). NCM460 and SW620 cells were cultured in DMEM medium (Gibco, USA) supplemented with 10% fetal bovine serum (Gibco, USA) and 1% penicillin–streptomycin (100 U/mL penicillin and 100 μg/mL streptomycin). HCT116 and HT29 cells were cultured in RPMI-1640 medium (Gibco, USA) supplemented with 10% fetal bovine serum (Gibco, USA) and 1% penicillin/streptomycin (Gibco). SLC35A3 overexpression models were established in HCT116 and SW620 cells using the pcDNA3.1( +)-SLC35A3 plasmid with the assistance of Lipofectamine 8000 transfection reagent (Beyotime). Empty vector was used as a negative control. After 48 h of cell culture, the corresponding phenotypic experiments were performed. The pcDNA3.1( +) plasmid containing SLC35A3 coding sequence was constructed by GenePharma (Shanghai, China). Primers for SLC35A3 (forward, 5′-GCTTGGTACCGAGCTCGGATCCG-3′, reverse, 5′-TGCTGGATATCT GCAGAATTCCTATGCTTTAGTGGGATTTCCTGCAGG-3′) were provided by GenePharma.

#### Quantitative real-time polymerase chain reaction (qRT-PCR)

The TRIzol reagent (ThermoFisher, CA, USA) was used to extract total RNA from cells and tissues. The PrimeScriptTM RT reagent kit (TaKaRa) was used for cDNA reverse transcription. qRT-PCR was performed using the Real-Time PCR System (Roche, Meylan, France) and SYBR Premix Ex TaqTM (TaKaRa). The primer sequences were as follows: SLC35A3 forward primer, 5′-CAGTGGCTGTCCCTAGTAATTTT-3′, SLC35A3 reverse primer, 5′-AGAACTGCCATGA GTCCTCA-3′. GAPDH forward primer, 5′-GAGCGAGATCCCTCAAAAT-3′, and GAPDH reverse primer, 5′-GGCTGTCATACTTCTCATGG-3′. The relative expression of SLC35A3 was calculated using the 2-∆∆Ct method.

#### Immunohistochemical (IHC) staining

We further verified the expression levels of SLC35A3 and CD4 proteins in tumor tissues and adjacent normal tissues of colorectal cancer patients through IHC. After deparaffinization, rehydration, and antigen retrieval by heating from the slides in sodium citrate buffer (pH 6.0), the antigens were retrieved. To inhibit endogenous peroxidase activity, they were blocked in 3% hydrogen peroxide for half an hour and then washed three times with PBS. Incubation with rabbit anti-SLC35A3 (SinoBiological, China) and rabbit anti-CD4 (Bioss, China) was performed overnight at 4 °C, followed by incubation with goat anti-rabbit IgG (TransGen Biotech, China) for 2 h at room temperature. After staining with 3,3′-diaminobenzidine (DAB), the sections were counterstained with hematoxylin, dehydrated, fixed, and sealed with a cover slip. The immunohistochemical results were analyzed in Image-Pro Plus 6.0, using the same brownish-yellow as the uniform standard for judging whether the image was positive. The cumulative optical density (IOD) and pixel area (area) of the tissue in each positive standard photo were analyzed, and the mean density was calculated as Mean density = IOD/area. The higher the Mean density value, the higher the expression level of the positive protein.

#### Western blotting

Western blot analysis was performed to evaluate the expression of SLC35A3 after transfecting HCT116 and SW620 cells. β-actin served as the internal control. Cells were lysed in ice-cold RIPA lysis buffer (Biosharp) with PMSF (Beyotime). Total protein quantification was performed using the BCA Protein Assay Kit (Huaxingbio). Proteins were separated by 10% SDS-PAGE (EpiZyme) and transferred to nitrocellulose membranes. The membranes were blocked at room temperature for 60 min with 1% bovine serum albumin. The primary antibody used was anti-SLC35A3 (Proteintech, 1:1000). The membranes were incubated overnight at 4℃ with the primary antibody, washed with Tris-buffered saline plus Tween, and then incubated at room temperature for 1 h with goat anti-rabbit secondary antibody (Huaxingbio, 1:10,000). Bands were developed using an Odyssey Imager.

#### CCK-8 assay

The effect of SLC35A3 overexpression on the viability of colorectal cancer cells was determined using the CCK-8 assay. HCT116 and SW620 cells were seeded in a 96-well plate (Corning). After cell attachment, cell viability was measured at 24 h, 48 h, and 72 h according to the manufacturer's instructions. The absorbance of cells was measured at 450 nm at three time points.

#### Cell invasion assay

The cell invasion assay was performed using 24-well Transwell chamber (Corning, USA). HCT116 and SW620 cells were added to Transwell chambers (BioCoat, 354,480) with a pore size of 0.8 mm to evaluate cell invasion. The chambers were filled with 600 μL of complete culture medium containing 10% FBS in the lower well of a 24-well plate. Then, 2 × 105 cells were seeded in the upper chambers and incubated in a cell culture incubator. After 24 h of incubation, non-migrating cells on the upper surface of the membrane were removed with a wet cotton swab. The invaded cells in the lower well were fixed with 4% paraformaldehyde solution for 30 min and stained with 0.1% crystal violet solution for 20 min. Images were captured under a microscope, and the invaded cells were counted and analyzed.

#### Cell apoptosis assay

Logarithmic phase HCT116 and SW620 cells were seeded in a 6-well plate at a density of 2 × 105 cells per well. After 24 h, cells were collected according to the instructions of the V-APC/7-AAD cell apoptosis detection kit (Elabscience, ECK-A218) and analyzed using a flow cytometer within 30 min.

#### Statistical analysis

We used R version 3.6.3 for bioinformatic analysis. The differential expression of SLC35A3 between normal and tumor tissues was analyzed using the Wilcoxon rank sum test and the paired t test. The correlation between SLC35A3 expression and clinicopathological features was analyzed using Fisher's exact test, chi-square test, Wilcoxon rank sum test, and logistic regression analysis. Receiver operating characteristic (ROC) curve analysis was used to evaluate the diagnostic accuracy, and the area under the curve (AUC) was calculated. Furthermore, Kaplan–Meier analysis and Cox regression analysis were used to evaluate the prognostic significance of SLC35A3 expression. In the Cox regression analysis, variables with statistical significance in the univariable Cox regression analysis were further included in the multivariable Cox model. Spearman test was used to analyze the correlation. The false discovery rate (FDR)-based adjusted *p*-value (FDR Q-value)was automatically evaluated. *p*-value < 0.05 was considered statistically significant.

## Results

### Expression of SLC35A3 in CRC patients

**Figure 1:**
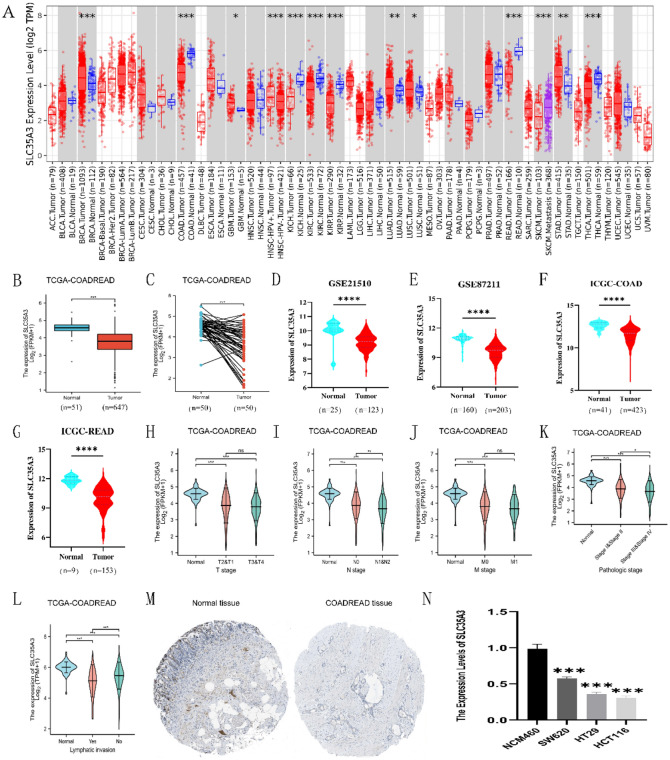
Expression of SLC35A3 in CRC analyzed from TIMER, TCGA, GEO, ICGC, HPA databases, and cell experiments. (**A**) Expression of SLC35A3 in different cancer types from TIMER database. (**B**) Significant decrease in SLC35A3 mRNA expression in CRC tumor tissue compared to normal tissue in TCGA-COADREAD dataset. (**C**) Significant decrease in expression of SLC35A3 mRNA in paired CRC tumor tissue compared to adjacent normal tissue in TCGA-COADREAD dataset. (**D**, **E**) Significantly lower expression of SLC35A3 mRNA in CRC tumor tissue compared to normal tissue in GSE21510 and GSE87211 datasets. (**F**, **G**) Significant decrease in expression of SLC35A3 mRNA in CRC tumor tissue compared to normal tissue in ICGC database. (**H**–**L**) Analysis of SLC35A3 mRNA expression based on T (**H**), N (**I**), M (**J**), pathological staging (**K**), and lymph node infiltration (**L**) in TCGA-COADREAD dataset. (**M**) Protein expression of SLC35A3 in normal colon tissue and colon cancer tissue based on HPA database. (**N**) Compared with normal colon epithelial cells NCM460, the expression level of SLC35A3 in colorectal cancer cells (including SW620, HT-29, HCT116) is reduced. Analysis was performed using the Wilcoxon rank sum test (**A**, **B**, **D**–**L**), the paired t test (C) and the ANOVA (N). ns denotes *p* > 0.05, *p < 0.05, **p < 0.01, ***p < 0.001 and ****p < 0.0001.

TIMER database was used to determine the overall expression level of SLC35A3 in different types of malignant tumors. The results showed that SLC35A3 had different expression patterns in various cancers. Compared to normal tissues, SLC35A3 mRNA expression was significantly decreased in colon adenocarcinoma (COAD) and rectal adenocarcinoma (READ) (Fig. 1A). To further determine the differential expression of SLC35A3 between colorectal tumors and normal tissues, we analyzed the TCGA-COADREAD dataset, which included RNA sequencing data and clinical information from 647 colorectal adenocarcinoma tissues and 51 normal colon tissues. The results showed that SLC35A3 was significantly downregulated in CRC tissues (*p* < 0.001, Fig. 1B). Additionally, we analyzed the expression levels of SLC35A3 in 50 pairs of CRC tissues and matched adjacent normal tissues, and found that SLC35A3 was significantly decreased in CRC tissues (*p* < 0.001, Fig. 1C). To validate these results, we downloaded microarray data (GSE21510 and GSE87211) from the GEO database. In addition, we retrieved colorectal cancer patient datasets (ICGC-COAD and ICGC-READ) from the ICGC database. The analysis results showed that SLC35A3 was significantly downregulated in colorectal adenocarcinoma compared to normal tissues (*p* < 0.0001, Fig. 1D-G). Immunohistochemical data from the HPA database also showed a significant decrease in SLC35A3 protein levels in CRC tissues (Fig. 1M).

### Low expression of SLC35A3 is associated with poor clinicopathological features of CRC

Table 1 shows that among the 647 colorectal cancer patients collected from the TCGA dataset, 644 had complete clinical and gene expression data. Based on the relative expression of SLC35A3 compared to the average expression value, CRC patients were divided into high expression group (n = 322) and low expression group (n = 322), and the correlation between SLC35A3 expression and various clinicopathological features of CRC patients was evaluated. The results showed that low expression of SLC35A3 mRNA was significantly associated with N stage (*p* = 0.004), pathological stage (*p* = 0.041), and lymph node invasion (*p* < 0.001), while it had no significant correlation with T stage, M stage, sex, age, CEA level, residual tumor, and neural invasion (*p* > 0.05).

The same results were observed in Fig. 1H–L, indicating that low expression of SLC35A3 was significantly associated with N stage (N0 vs N1/N2, *p* < 0.01, Fig. 1I), pathological stage (stage I/II vs III/IV, *p* < 0.05, Fig. 1K), and lymph node invasion (yes vs no, *p* < 0.001, Fig. 1L). However, SLC35A3 mRNA expression had no significant correlation with T stage (T1/T2 vs T3/T4, *p* > 0.05, Fig. 1H) and M stage (M0 vs M1, *p* > 0.05, Fig. 1J).

Additionally, the results of univariable logistic regression analysis (Table 2) showed that SLC35A3 mRNA expression was closely related to N stage (OR = 0.623, 95% confidence interval (CI): 0.454–0.853, *p* = 0.003), pathological stage (OR = 0.630, 95%CI 0.457–0.865, *p* = 0.004), and lymphatic infiltration (OR = 2.090, 95%CI 1.493–2.938, *p* < 0.001).

**Table 2:** Logistic regression analysis of the correlation between SLC35A3 expression and clinical pathological parameters in colorectal cancer.

Characteristics	Total(N)	OR (95% CI)	P value
T stage (T3&T4 vs. T1&T2)	641	0.782 (0.531–1.148)	0.211
N stage (N1&N2 vs. N0)	640	0.623 (0.454–0.853)	**0.003**
M stage (M1 vs. M0)	564	0.777 (0.490–1.223)	0.277
Pathologic stage (Stage III&Stage IV vs. Stage I&Stage II)	623	0.630 (0.457–0.865)	**0.004**
Gender (Male vs. Female)	644	0.916 (0.672–1.249)	0.580
Age (< = 65 vs. > 65)	644	0.975 (0.713–1.332)	0.873
CEA level (< = 5 vs. > 5)	415	0.928 (0.622–1.382)	0.712
Residual tumor (R1&R2 vs. R0)	510	0.933 (0.492–1.757)	0.829
Perineural invasion (No vs. Yes)	235	1.140 (0.630–2.054)	0.663
Lymphatic invasion (No vs. Yes)	582	2.090 (1.493–2.938)	** < 0.001**
Colon polyps present (No vs. Yes)	323	1.076 (0.668–1.730)	0.762

*CEA* Carcinoembryonic antigen. *OR* Odds Ratio, *CI* 95% confidence interval. The statistical significance values are displayed in bold (*p* < 0.05).

### SLC35A3 is downregulated in colon cancer tissue compared to adjacent normal tissue

**Figure 2:**
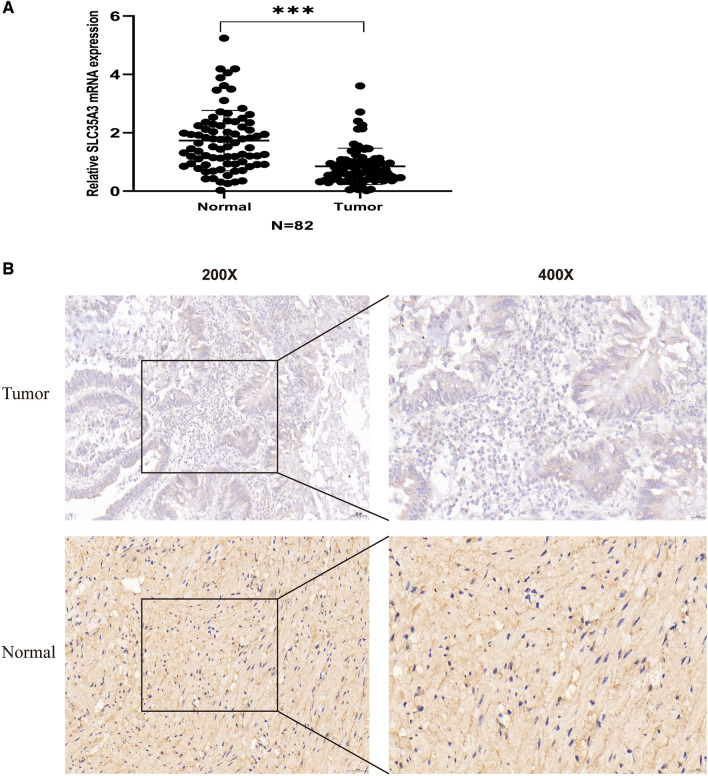
Decreased expression of SLC35A3 in clinical CRC tissue. (**A**) qRT-PCR analysis of cancer tissue and adjacent normal tissue samples from CRC patients. (**B**) Representative images showing protein expression of SLC35A3 in paraffin-embedded CRC tissue and adjacent normal tissue. Analysis was performed using the paired t test and ***p < 0.001.

qRT-PCR was used to detect the expression levels of SLC35A3 in human normal colon epithelial cell line NCM460 and human colon cancer cell lines HCT116, HT-29, and SW620. Compared to normal colon epithelial cells, colon cancer cells (SW620, HT29, HCT116) exhibited significant downregulation of SLC35A3 mRNA expression (Fig. 1N). qRT-PCR and immunohistochemistry were used to detect the expression of SLC35A3 in 82 pairs of CRC tissues and their adjacent normal tissues. Compared to adjacent normal tissue, both mRNA (Fig. 2A) and protein (Fig. 2B) expression levels of SLC35A3 were significantly downregulated in CRC tissue. These results suggest that SLC35A3 may be involved in the occurrence and development of cancer in CRC patients.

### Low expression of SLC35A3 predicts poor prognosis in CRC patients

**Figure 3:**
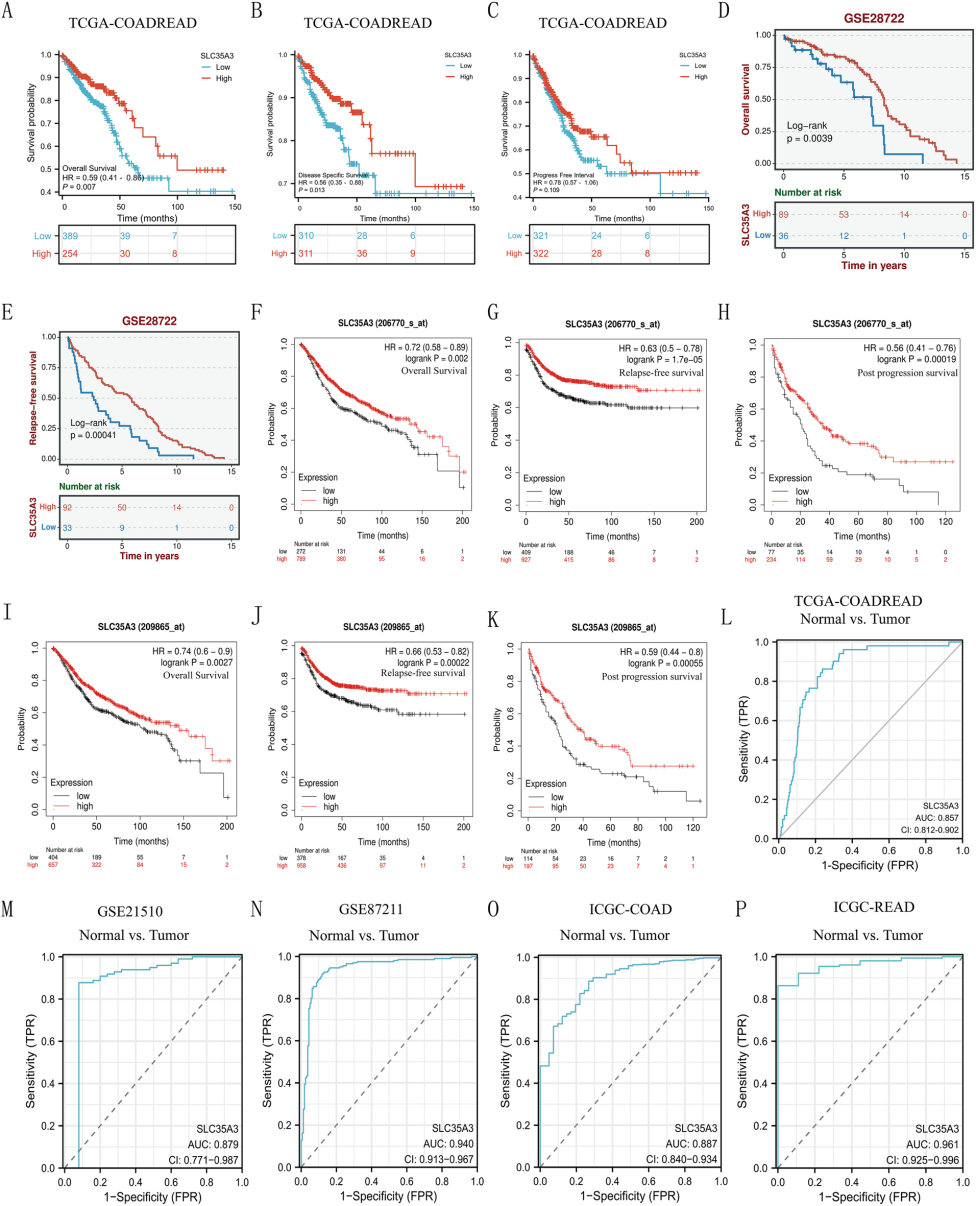
Prognostic and Diagnostic value of SLC35A3 in CRC. (**A**–**C**) Survival curves from TCGA-CADREAD dataset showing OS, PFI and DSS of patients with low or high expression of SLC35A3. (**D**, **E**) Analysis of GSE28782 dataset showing better OS and RFS in patients with high expression of SLC35A3 compared to those with low expression. (**F**–**K**) Further validation of clinical data from CRC patients in Kaplan–Meier Plotter database, demonstrating significantly better OS, RFS, and PPS in patients with high expression of SLC35A3 compared to those with low expression. (**L**–**P**) ROC analysis based on CRC datasets from TCGA, GEO, and ICGC databases, indicating accurate discrimination between CRC tumor tissue and normal tissue based on SLC35A3 expression; *AUC* area under the curve, *OS* overall survival, *DSS* disease-specific survival, *PFI* progression-free interval, *RFS* relapse-free survival, *PPS* post-progression survival, *CRC* colorectal cancer, *T* tumor distribution, *N* lymph node metastasis, *M* distant metastasis. *CI* 95% confidence interval. Analysis was performed using the Cox regression analysis (**A**–**C**) and the Log-rank test (**D**–**K**).

Based on the TCGA-COADREAD dataset, Kaplan–Meier survival analysis was performed to validate the correlation between SLC35A3 expression and prognosis. Low expression of SLC35A3 was positively associated with poor overall survival (OS) (HR = 0.62, 95% CI = 0.44–0.88, *p* = 0.008, Fig. 3A). Similarly, decreased expression of SLC35A3 was significantly correlated with disease-specific survival (DSS) (HR = 0.59, 95% CI = 0.37–0.93, *p* = 0.022, Fig. 3B). However, low expression of SLC35A3 was not associated with poor progression-free interval (PFI) (HR = 0.79, 95% CI = 0.58–1.07, *p* = 0.132, Fig. 3C). Based on the GSE28722 dataset, the results showed that patients with high SLC35A3 expression had better OS and RFS than those with low SLC35A3 expression (Fig. 3D, E). The clinical data of colorectal cancer patients from the KM-plotter database further verified that patients with high SLC35A3 expression had significantly better OS, RFS, and PPS than those with low SLC35A3 expression (Fig. 3F–K). In the univariable analysis, T stage, N stage, M stage, pathological stage, age, SLC35A3, and CEA expression levels influenced the prognosis of CRC patients (all *p* < 0.05). Furthermore, multivariable Cox regression showed that M stage, pathological stage, age, and SLC35A3 expression level were independent risk factors for poor overall survival (OS) in CRC patients (Table 3).

**Table 3:** Univariable and multivariable analysis of clinical pathological factors related to OS in CRC patients.

Characteristics	Total (N)	Univariable analysis		Multivariable analysis	
		Hazard ratio (95% CI)	*p* value	Hazard ratio (95% CI)	*p* value
T stage (T1/T2 vs. T3/T4)	640	2.468 (1.327–4.589)	**0.004**	1.900 (0.655–5.516)	0.238
N stage (N0 vs. N1/N2)	639	2.627 (1.831–3.769)	** < 0.001**	0.391 (0.114–1.346)	0.137
M stage (M0 vs. M1)	563	3.989 (2.684–5.929)	** < 0.001**	2.250 (1.066–4.751)	**0.033**
Pathologic stage (Stage I/Stage II ) vs. (Stage III/Stage IV)	622	2.988 (2.042–4.372)	** < 0.001**	5.909 (1.371–25.477)	**0.017**
Gender (Female vs. Male)	643	1.054 (0.744–1.491)	0.769		
CEA level (≤ 5 vs. > 5)	414	2.620 (1.611–4.261)	** < 0.001**	1.506 (0.800–2.832)	0.204
SLC35A3 ( High vs. Low)	643	0.620 (0.437–0.882)	**0.008**	0.486 (0.276–0.855)	**0.012**
Age (≤ 65 vs. > 65)	643	1.939 (1.320–2.849)	** < 0.001**	4.067 (2.101–7.872)	** < 0.001**

*CRC* colorectal cancer, *OS* overall survival, *CI* confidence in interval, *T* topography distribution, *N* lymph node metastasis, *M* distant metastasis. Statistically significant values are shown in bold (*p* < 0.05).

### SLC35A3 expression as a potential diagnostic biomarker in CRC

ROC analysis was conducted to evaluate the potential value of SLC35A3 as a diagnostic biomarker for differentiating CRC tissues from normal tissues in the TCGA-COADREAD, GSE87211, GSE21510, ICGC-COAD, and ICGC-READ datasets. The results showed that SLC35A3 could serve as a good diagnostic biomarker for CRC patients (Fig. 3L–P).

### Construction and validation of nomogram based on the expression level of SLC35A3

**Figure 4:**
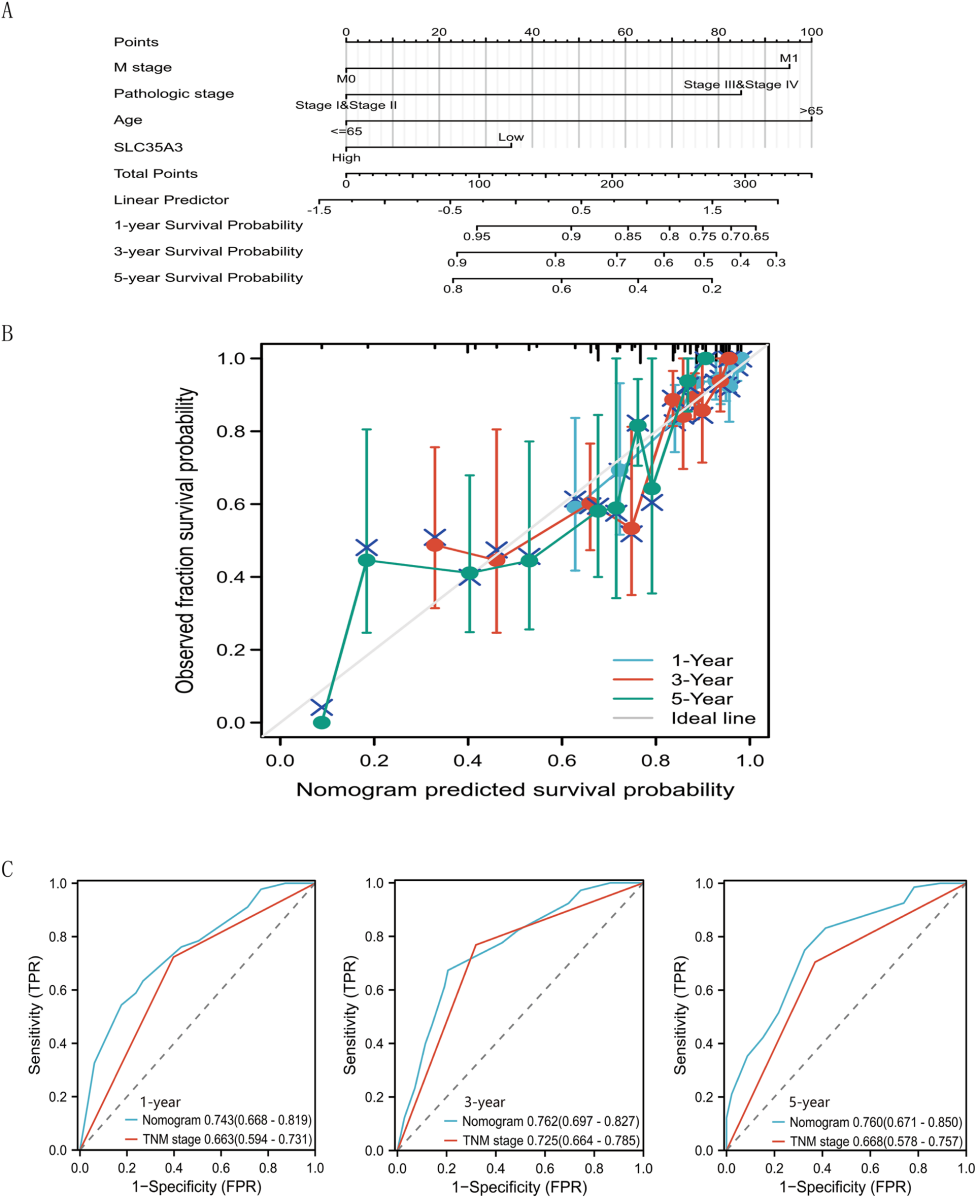
Construction and validation of column charts based on SLC35A3 expression levels. (**A**) The nomograph for predicting OS probability of CRC patients at 1, 3, and 5 years. (**B**) A nomograph calibration map that predicts OS possibilities. (**C**) Nomogram demonstrating the prognostic value of SLC35A3 in CRC, which exceeds that of TNM staging. OS: overall survival.

A nomogram was developed to assist clinicians in determining the prognosis of CRC patients. The nomogram was based on the clinical features (M stage, pathological stage, age, SLC35A3) that were independently associated with patient survival in the multivariable analysis (Fig. 4A). The concordance index of the nomogram was 0.738. The calibration plot for validating the predictive model reliability is shown in Fig. 4B. The bias-corrected line in the calibration plot is close to the ideal curve (also known as the 45-degree line), indicating good consistency between the observed and predicted values. These results suggest that the nomogram is a model superior to a single prognostic factor for determining the long-term survival rate (1, 3, and 5 years) of CRC patients. Furthermore, we compared the prognostic value of our constructed nomogram with the TNM stage for CRC patients, and the results showed that the AUC values of our constructed nomogram were higher than those of the TNM stage at 1-year, 3-year, and 5-year (Fig. 4C). These data suggest that our nomogram has a higher prognostic value in colorectal cancer compared to the TNM stage.

### SLC35A3 mutations in CRC

**Figure 5:**
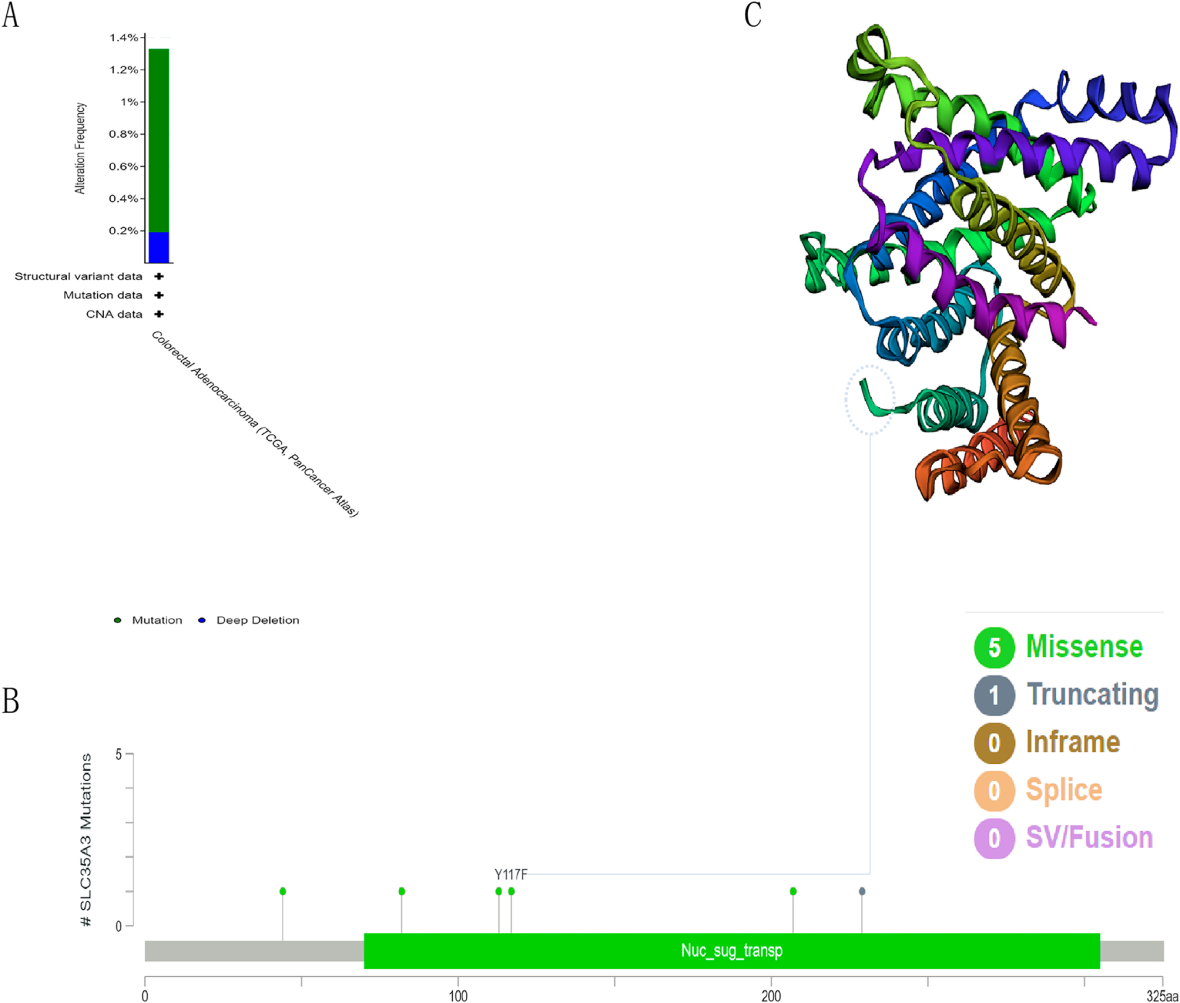
SLC35A3 mutations in CRC. (**A**, **B**) The cBioPortal tool was used to displays the mutation frequency (**A**) and mutation sites (**B**) of SLC35A3 in CRC. (**C**) Three-dimensional protein structure of SLC35A3.

The accumulation of genetic mutations promotes the development of cancer. In order to explore the mutations of the SLC35A3 gene in CRC, we analyzed its mutation status based on TCGA data using the cBioPortalTM platform. The results showed that the mutation rate of SLC35A3 in CRC was 1.3% (Fig. 5A). Missense mutations and truncating mutations were the main types of mutations in SLC35A3 (Fig. 5B). In CRC cases, a missense mutation Y117F was detected in the Nuc_sug_transp domain, and the three-dimensional structure of the SLC35A3 protein showed the Y117F mutation in Fig. 5C. Other mutations, including V44A, M82T, Y113N, R207S, and E229, were detected in the SLC35A3 protein structure in other CRC patients. These findings suggest that changes in the SLC35A3 gene may play a key role in the pathogenesis of CRC.

### Methylation levels of SLC35A3 promoter in CRC

**Figure 6:**
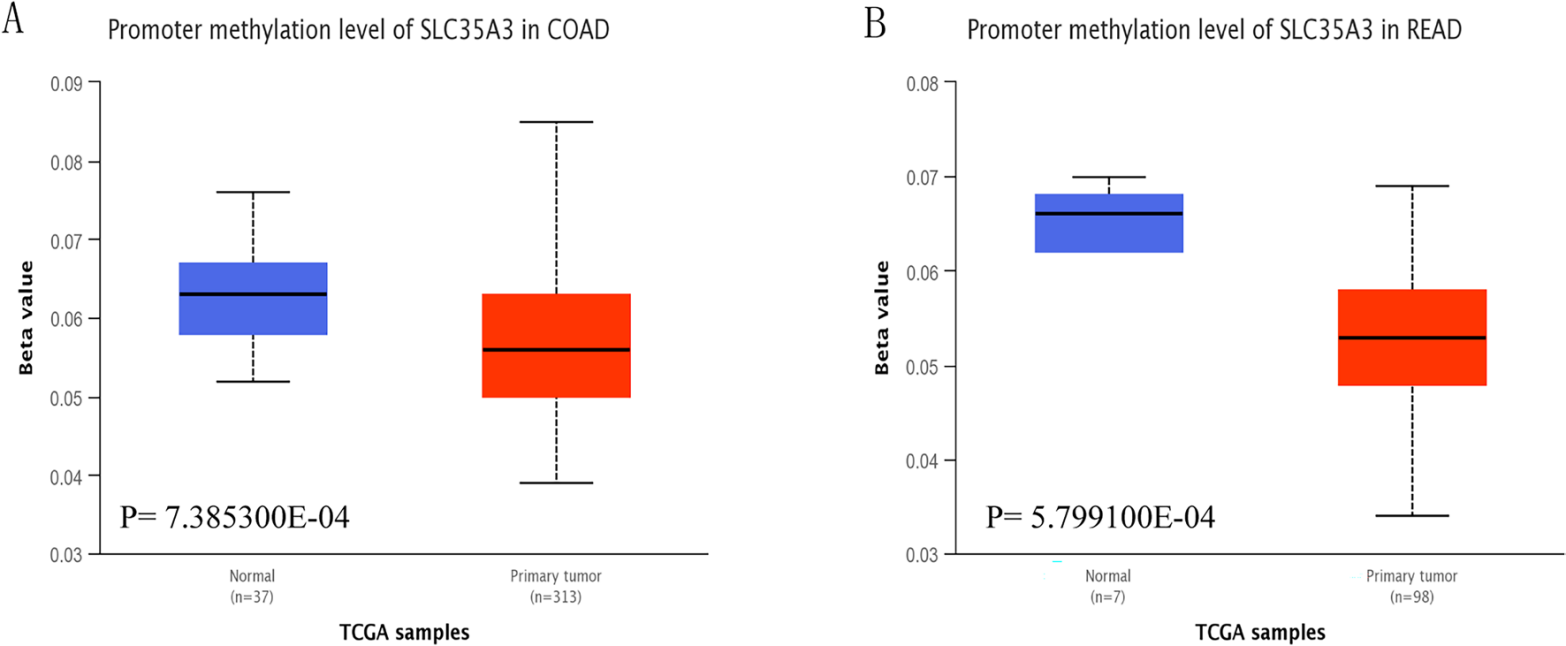
The promoter methylation level of SLC35A3 in colorectal cancer tissues and adjacent normal tissues. UALCAN tool was used to analyze the promoter methylation level of SLC35A3 gene in colon cancer and its adjacent normal tissues (**A**) and rectal cancer and its adjacent normal tissues (**B**).

Promoter DNA methylation has been shown to affect transcriptional repression and contribute to tumorigenesis^33^. We compared the methylation levels of the SLC35A3 promoter in colorectal cancer and adjacent normal tissues. Our analysis showed a significant decrease in the methylation levels of the SLC35A3 promoter in colon cancer (Fig. 6A) and cancer (Fig. 6B), with statistical significance. These results suggest that the reduced expression of SLC35A3 in CRC may be due to changes in promoter methylation.

### Potential biological functions and pathways of SLC35A3 in CRC

**Figure 7:**
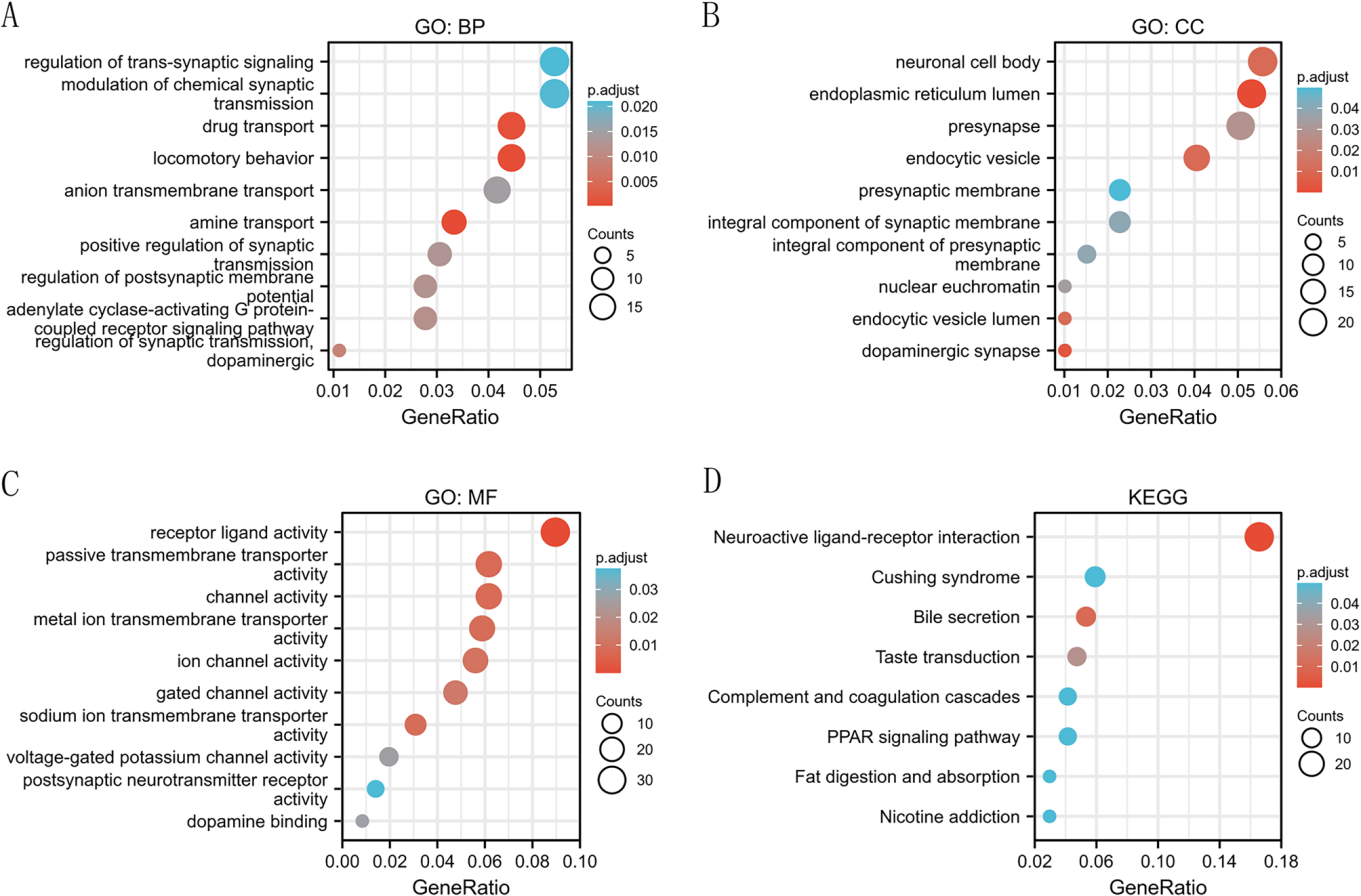
Go and KEGG enrichment analysis of differential genes in CRC tissues of low SLC35A3 group and high SLC35A3 group. (**A**–**C**) GO enrichment analysis showed that BP (biological process), CC (cellular component) and MF (molecular function) of differential genes in low SLC35A3 group and high SLC35A3 group. (**D**) KEGG metabolic pathway enriched by different genes in low SLC35A3 group and high SLC35A3 group.

We analyzed the potential biological functions of SLC35A3 in CRC and performed enrichment analysis on differentially expressed genes (|logFC|> 1, P.adj < 0.05) between the low SLC35A3 group and the high SLC35A3 group. GO biological process analysis showed significant enrichment of metal ion transmembrane transport, drug transport, locomotion, positive regulation of synaptic transmission, and adenylate cyclase activated G protein coupled receptor signal pathway (Fig. 7A). GO cellular component analysis showed significant enrichment of endoplasmic reticulum lumen, presynaptic membrane, postsynaptic membrane, dopamine synapse, and more (Fig. 7B). Molecular function analysis showed significant enrichment of receptor-ligand activity, channel activity, metal ion transmembrane transporter activity, and passive transmembrane transporter activity (Fig. 7C). KEGG analysis revealed that the interaction of neuroactive ligand-receptor was the most significantly enriched pathway (Fig. 7D). In summary, the study results indicate that SLC35A3 may be involved in the changes of cell membrane potential, transmembrane transporter protein activity, cell communication, and the "neuroactive ligand-receptor interaction" pathway, thereby regulating the proliferation and invasion of CRC.

**Figure 8:**
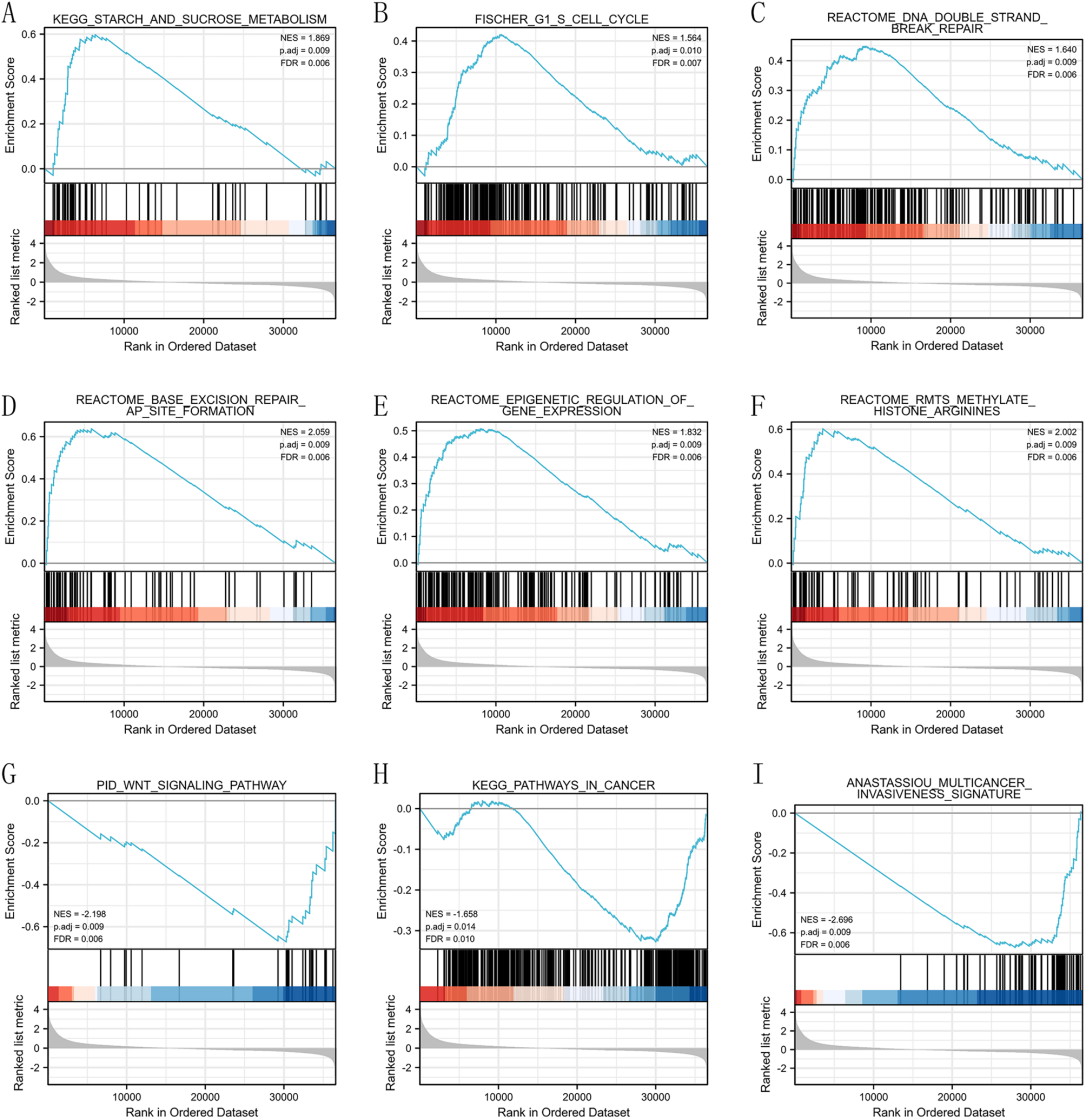
Enrichment results of gene set enrichment analysis (GSEA). (**A**) Starch and sucrose metabolism pathway, (**B**) G1, S cell cycle, (**C**) DNA double strand break repair, (**D**) base excision repair, (**E**) epigenetic regulation of gene expression, (**F**) histone arginine methylation, (**G**) WNT signal pathway, (**H**) cancer pathway, and (**I**) multiple cancer invasive characteristics were significantly enriched in SLC35A3-related CRC. NES, normalized enrichment fraction; FDR, false discovery rate.

In addition, GSEA was performed based on normalized enrichment score (NES) and false discovery rate (FDR, q-value) to elucidate the potential biological pathways regulated by SLC35A3 between the high expression group and the low expression group. As shown in Fig. 8 and Table 4, several biological pathways were significantly enriched in the SLC35A3 overexpression group, including starch and sucrose metabolism pathway (Fig. 8A), cell cycle G1/S phase (Fig. 8B), DNA double-strand break repair (Fig. 8C), base excision repair (Fig. 8D), gene expression epigenetic regulation (Fig. 8E), and histone arginine methylation (Fig. 8F). In addition, the WNT signaling pathway (Fig. 8G) and cancer pathway (Fig. 8H) were significantly enriched in the SLC35A3 low expression group in various cancer invasion features (Fig. 8I). These results suggest that SLC35A3 may affect the progression of CRC through the regulation of energy metabolism, cell cycle, DNA repair, epigenetic regulation of gene expression, and carcinogenesis pathways.

**Table 4:** Results of Gene Set Enrichment Analysis (GSEA).

Description	Set size	EnrichmentScore	NES	*p* value	p.adjust	q values	Rank
KEGG_STARCH_AND_SUCROSE_METABOLISM	52	0.59773391	1.86860419	0.00040855	0.0085906	0.0061857	6357
REACTOME_BASE_EXCISION_REPAIR_AP_SITE_FORMATION	63	0.63736182	2.058913	0.00013193	0.0085906	0.0061857	5890
REACTOME_RMTS_METHYLATE_HISTONE_ARGININES	76	0.602562409	2.00222025	0.00012820	0.00859059	0.00618570	3941
REACTOME_EPIGENETIC_REGULATION_OF_GENE_EXPRESSION	146	0.50738425	1.83162574	0.00011663	0.00859059	0.00618570	8157
REACTOME_DNA_DOUBLE_STRAND_BREAK_REPAIR	166	0.44863791	1.64049479	0.00068564	0.00863341	0.00621653	9366
FISCHER_G1_S_CELL_CYCLE	198	0.4203582	1.56352259	0.00122754	0.01016656	0.00732048	10,519
PID_WNT_SIGNALING_PATHWAY	27	− 0.673145376	− 2.1977280	0.00029832	0.00859059	0.00618570	6273
ANASTASSIOU_MULTICANCER_INVASIVENESS_SIGNATURE	64	− 0.674435264	− 2.6959558	0.00041528	0.00859059	0.00618570	8553
KEGG_PATHWAYS_IN_CANCER	325	− 0.32795732	− 1.6580184	0.00205761	0.01397440	0.01006233	6564

*NES (normalized enrichment score)* Corrected and normalized enrichment score. *p.adjust* Corrected *p*-values obtained through p-value adjustment methods. *q value* The adjusted *p*-value after multiple hypothesis testing correction.

#### Correlation between SLC35A3 expression and immune infiltration levels in CRC

**Figure 9:**
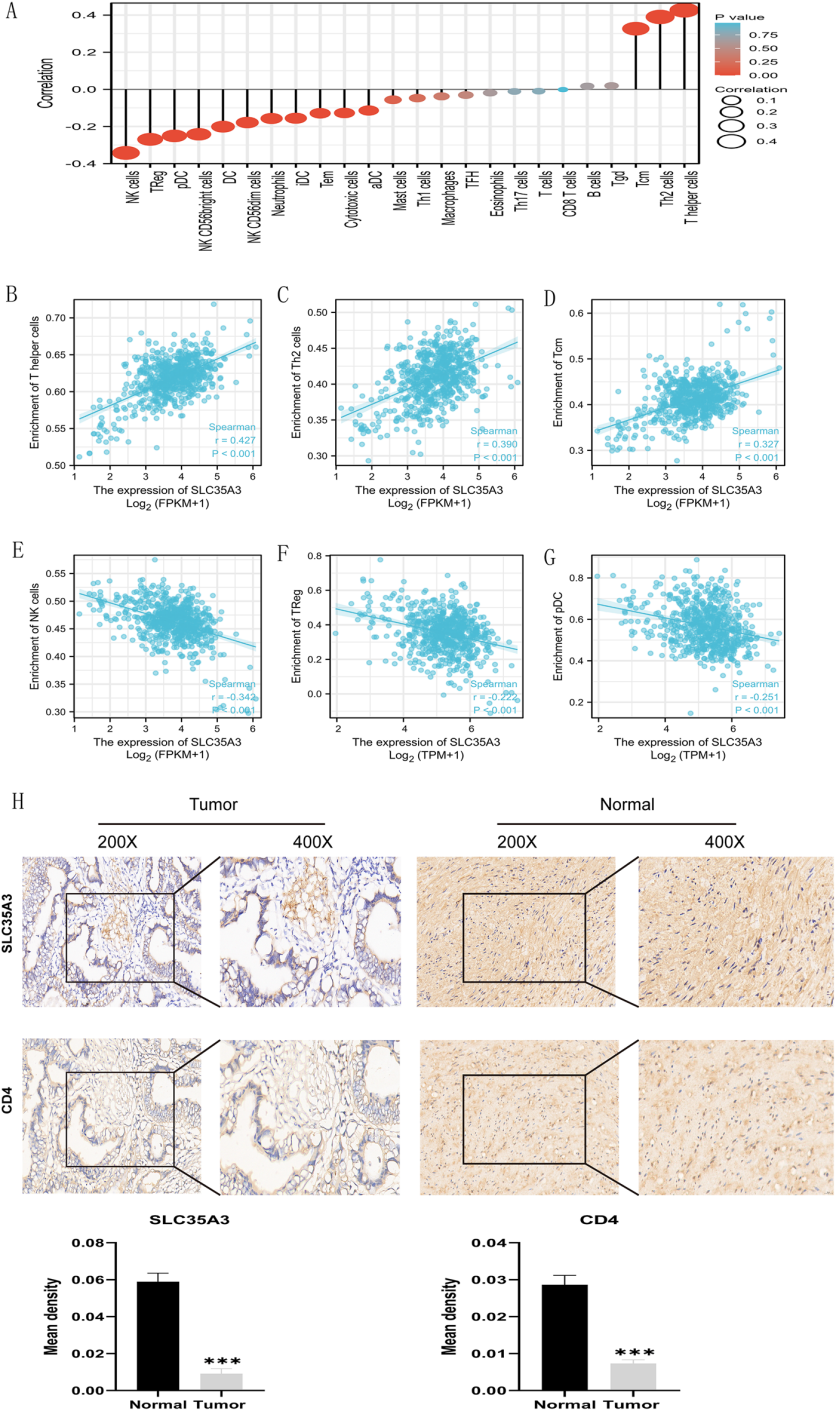
Correlation analysis between SLC35A3 expression and immune infiltration. (**A**) Relationship between SLC35A3 expression and infiltration of tumor-infiltrating lymphocytes in 24 cases. (**B**–**G**) Correlation between SLC35A3 expression and immune infiltration levels of T helper cells (**B**), Th2 cells (**C**), Tcm cells (**D**), NK cells (**E**), Treg cells (**F**), and pDC cells (**G**). (**H**) Immunohistochemistry results showing downregulation of SLC35A3 and CD4 expression in colon cancer tissue compared to adjacent normal tissue. Spearman correlation analysis (**A**–**G**) and the paired t test (H) was conducted, *** *p* < 0.001.

Tumor infiltrating lymphocytes are closely related to the improvement of cancer prognosis^31,34^. Therefore, we further explored the relationship between SLC35A3 expression and CRC immune infiltration. The correlation between SLC35A3 and 24 immune cell subsets in CRC was analyzed using Spearman r's ssGSEA (Fig. 9A). We found a positive correlation between SLC35A3 and T helper cells (R = 0.427, *p* < 0.001, Fig. 9B), Th2 cells (R = 0.390, *p* < 0.001, Fig. 9C), and Tcm cells (R = 0.327, *p* < 0.001, Fig. 9D) in CRC. Additionally, SLC35A3 expression was negatively correlated with NK cells (R = -0.342, *p* < 0.001, Fig. 9E), Treg cells (R =  − 0.1222, *p* < 0.001, Fig. 9F), pDC cells (R =  − 0.551, *p* < 0.001, Fig. 9G), NK CD56bright cells (R =  − 0.242, *p* < 0.001, data not shown), NK CD56dim cells (R =  − 0.179, *p* < 0.001, data not shown), cytotoxic cells (R =  − 127, *p* = 0.001, data not shown), and Tem cells (R =  − 0.129, *p* = 0.001, data not shown). Furthermore, we validated the correlation between SLC35A3 and T helper cells infiltration. The immunohistochemical results showed that the surface marker CD4 expression of T helper cells was also upregulated in normal tissues with high SLC35A3 expression (Fig. 9H).

#### Association of SLC35A3 expression with immune checkpoint (ICP) genes in CRC.

**Figure 10:**
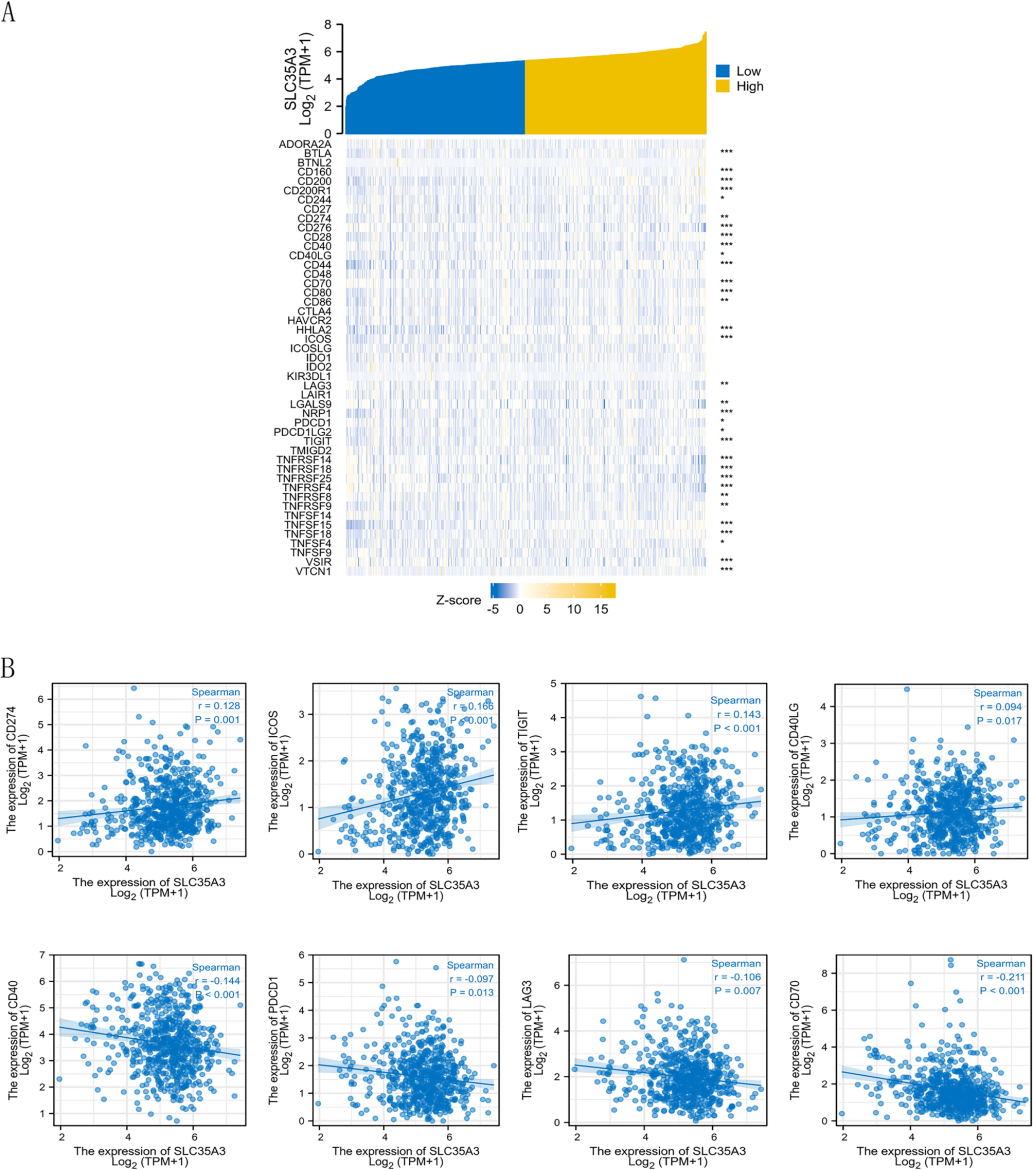
Correlation analysis between SLC35A3 and immune checkpoints based on the TCGA-COADREAD dataset. (**A**) Correlation heatmap between SLC35A3 expression and immune checkpoint. (**B**) The expression of SLC35A3 is associated with 8 immune checkpoints (CD274, ICOS, TIGIT, CD40LG, CD40, PDCD1, LAG3, CD70). Spearman correlation analysis was conducted.

Immune checkpoint (ICP) genes have a significant impact on immune cell infiltration and immune therapy^35^. We further investigated the relationship between SLC35A3 expression and ICP genes in CRC to explore the potential of SLC35A3 in immunotherapy. The results showed that SLC35A3 expression was closely associated with most of the 47 ICP genes (Fig. 10A). It was positively correlated with CD274 (R = 0.128, *p* < 0.001), ICOS (R = 0.166, *p* < 0.001), TIGIT (R = 0.143, *p* < 0.01), and CD40LG (R = 0.094, *p* < 0.017). It was negatively correlated with CD40 (R = -0.144, *p* < 0.0014), PDCD1 (R =  − 0.097, *p* = 0.013), LAG3 (R =  − 0.106, *p* = 0.007), and CD70 (R =  − 0.211, *p* < 0.001) (Fig. 10B).

#### Cell experiments to validate the effect of SLC35A3 on the biological behaviors of colorectal cancer cells

**Figure 11:**
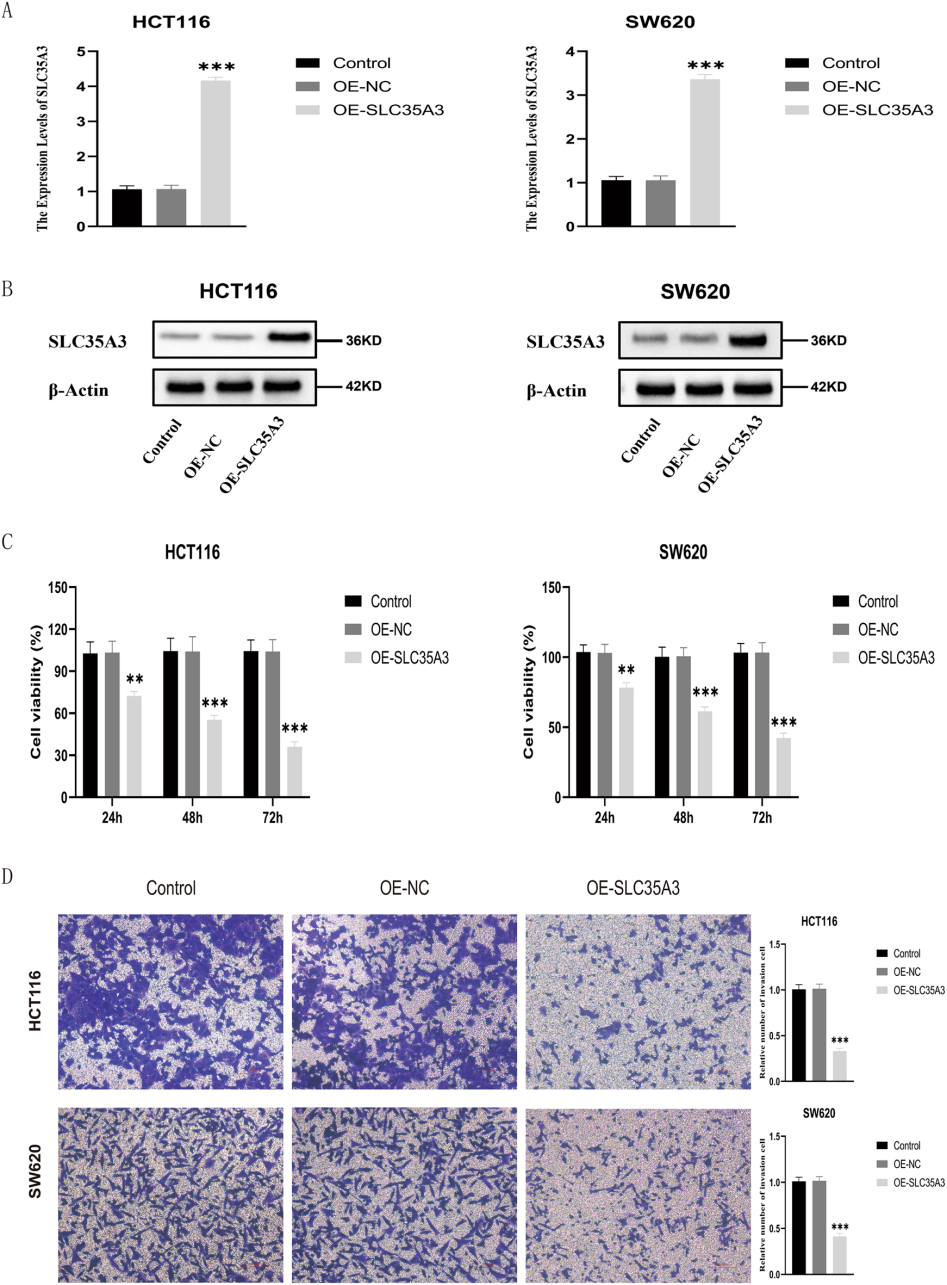
Overexpression of SLC35A3 significantly inhibits proliferation and invasion of colorectal cancer cells (HCT116 and SW620). (**A**, **B**) After plasmid transfection, significant upregulation of SLC35A3 mRNA (**A**) and protein (**B**) levels in HCT116 and SW620 cells of OE-SLC35A3 group. (**C**, **D**) Overexpression of SLC35A3 significantly suppresses the proliferation (**C**) and invasion (**D**) abilities of HCT116 and SW620 cells. Three biological replicates were performed, and the results were subjected to statistical analysis. Analysis was performed using the ANOVA (**A**, **C**, **D**), **p < 0.01, ***p < 0.001.

**Figure 12:**
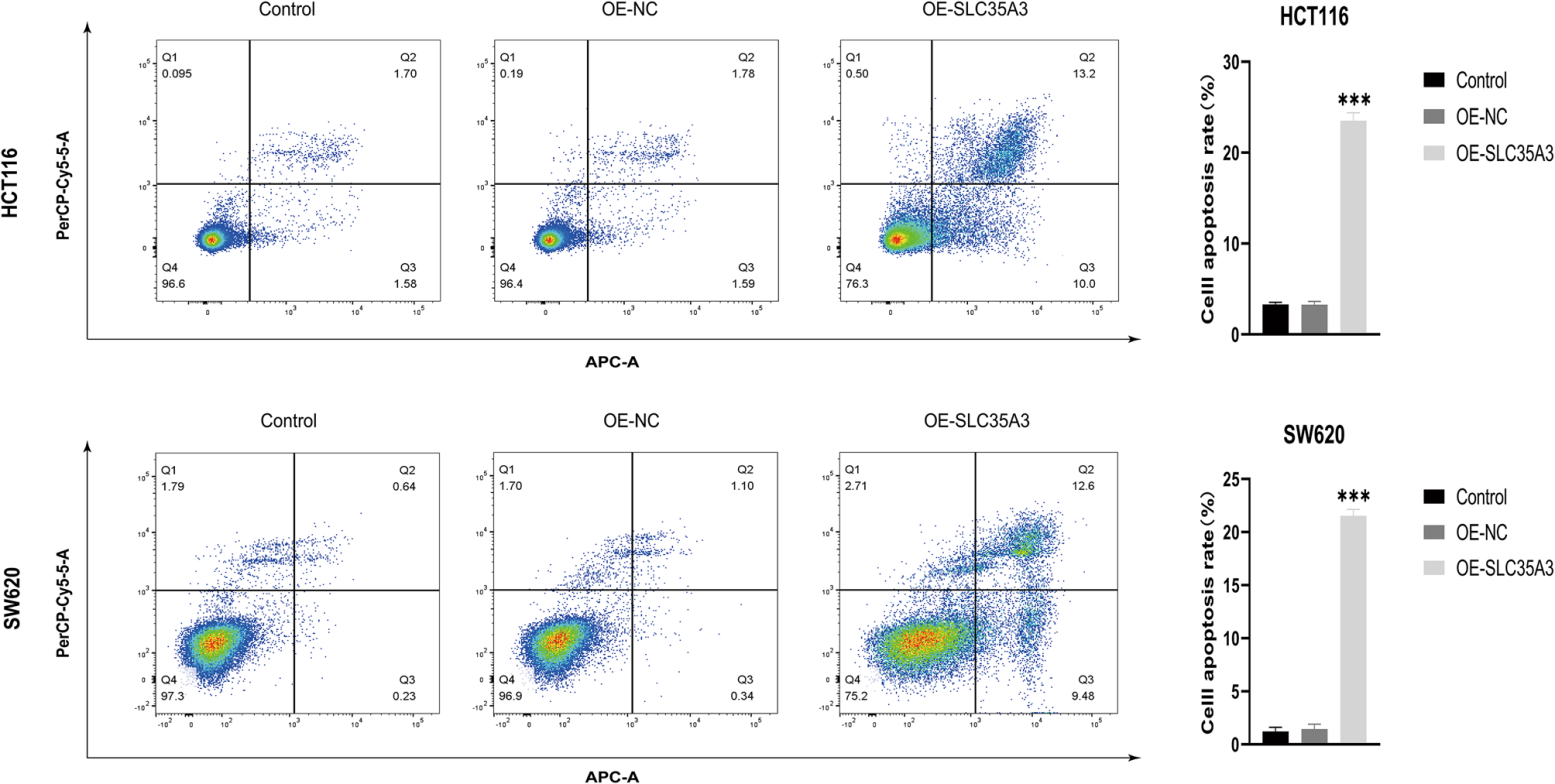
Overexpression of SLC35A3 significantly promotes apoptosis of colorectal cancer cells (HCT116 and SW620). Three biological replicates were performed, and the results were subjected to statistical analysis. Analysis was performed using the ANOVA, ***p < 0.001.

To further study the role of SLC35A3 in colorectal cancer cells, we overexpressed SLC35A3 using overexpression plasmids (OE-SLC35A3) in colorectal cancer cell lines HCT116 and SW620. Non-transfected cells and empty vector transfected cells (OE-NS) were used as negative controls. In both cell lines, there was no difference in SLC35A3 mRNA and protein levels between the two control groups, but the mRNA and protein levels of SLC35A3 were significantly upregulated in the OE-SLC35A3 group (Fig. 11A, B) (Supplementary Information). CCK-8 cell proliferation assays showed that the proliferation ability of cells in the OE-NC group did not change significantly compared to the control group, while the proliferation ability of cells in the OE-SLC35A3 group was significantly reduced (Fig. 11C). Transwell invasion assays showed that overexpressing SLC35A3 significantly inhibited the invasion ability of HCT116 and SW620 cells (Fig. 11D). Furthermore, cell apoptosis assays showed that overexpressing SLC35A3 significantly increased the apoptosis rate of HCT116 and SW620 cells (Fig. 12).

## Discussion

Early symptoms of CRC are not obvious. Due to the lack of effective diagnostic methods, most cancer patients are already in the advanced stage with synchronous metastasis and cannot undergo curative surgery, resulting in poor prognosis^36,37^. Traditional biomarkers such as carbohydrate antigen 19-9 (CA19-9) and carcinoembryonic antigen (CEA) have low sensitivity in the diagnosis of CRC, and their role in early diagnosis of CRC is not satisfactory^38^. Therefore, it is necessary to find more effective early diagnostic molecules and new therapeutic targets, and establish an effective prevention, early diagnosis, and treatment system.

Solute Carrier 35 (SLC35) is a subfamily of nucleotide sugar transporters (NST) that transports nucleotide sugars between cells and plays important roles in cell recognition, regulation, signal transduction, immune response, cell transformation, and the development of diseases. In recent years, numerous studies have focused on analyzing the role of the SLC35 family in diseases including cancer. Xu recently comprehensively analyzed the clinical significance and immunotherapeutic value of 35 members of the SLC35 family in 33 human cancers^39^. Previous studies have reported the important role of SLC35A3 in the development of T-cell lymphoblastic lymphoma, pancreatic cancer, and breast cancer, and it can serve as an early warning marker for these cancers. However, there have been no reports on the clinical significance of SLC35A3 in CRC, which requires further exploration.

TIMER database analysis showed that the expression level of SLC35A3 mRNA varies in different types of cancer. In order to understand the role of SLC35A3 in the development of colorectal cancer (CRC), we evaluated the expression level of SLC35A3 in tumor tissues and adjacent normal tissues of CRC based on the TCGA, GEO, ICGC, and HPA databases. The results showed that SLC35A3 was downregulated in CRC compared to normal tissues. We then detected the expression in normal colonic mucosal cells (NCM460) and CRC cells (SW620, HCT116, and HT29), and found that SLC35A3 mRNA level was significantly decreased in CRC cells compared to normal colonic epithelial cells (*p* < 0.001). Furthermore, we verified the mRNA and protein levels of SLC35A3 in clinical samples of CRC and adjacent normal tissues through qRT-PCR and immunohistochemistry experiments. The results showed that SLC35A3 mRNA and protein levels were decreased in CRC tumor tissues compared to adjacent normal tissues. These data suggest that the decreased expression of SLC35A3 may be associated with poor prognosis in CRC.

We further explored the correlation between SLC35A3 expression and prognosis of CRC patients. The analysis of the TCGA-COADREAD dataset showed that downregulation of SLC35A3 expression was significantly associated with worse overall survival (OS) and disease-specific survival (DSS) in CRC patients. The analysis of the GSE28722 dataset and the Kaplan–Meier Plotter tool further validated the significant association between high expression of SLC35A3 and better OS, relapse-free survival (RFS), and post progression survival (PPS) in CRC patients. It is worth noting that the downregulation of SLC35A3 expression was positively correlated with adverse clinical pathological features (N stage, pathological stage, and lymph node invasion) of CRC. The ROC curve analysis based on the TCGA-COADREAD, GSE21510, GSE87211, and ICGC databases all indicated that SLC35A3 is a potential diagnostic biomarker for distinguishing CRC tissues from normal tissues. Univariable and multivariable Cox analyses both demonstrated that SLC35A3 is an independent prognostic factor for CRC patients. These results suggest that SLC35A3 may play an important role in inhibiting the proliferation and metastasis of CRC. In addition, in order to improve the predictive ability of prognosis in CRC patients, we constructed a nomogram prognostic model based on the expression level of SLC35A3, and compared its predictive efficacy with TNM staging. The results showed that the AUC values of our constructed nomogram prognostic model were higher than those of TNM staging at 1, 3, and 5 years, indicating that our nomogram may have higher predictive value in the prognosis of colorectal cancer patients.

Spontaneous genetic mutations can accumulate in somatic cells throughout a person’s life. Although most mutations have no apparent effect on individuals, some mutations can alter critical cellular functions. The accumulation of mutations can lead to cancer and aging^40^. Many mutated genes have been reported to be associated with the occurrence and prognosis of CRC^41–44^. NCCN guidelines recommend the evaluation of tumor gene status, including KRAS/NRAS and BRAF mutations, as well as HER2 amplification and microsatellite instability (MSI)/mismatch repair (MMR) status, for the systemic treatment of advanced or mCRC^45^. Therefore, studying the genetic mutations in CRC patients to determine their impact on the prognosis and treatment of CRC is of great significance. In this study, we found that the mutation frequency of SLC35A3 in CRC was 1.3%, with missense mutations and truncating mutations being the main mutation types. DNA methylation plays an important role in gene expression regulation^46^, cell development and lineage specification^47^, X-chromosome inactivation^48^, genomic imprinting^49^, tissue differentiation^50^, aging^51^, and other biological functions. Characteristic changes in DNA methylation have been confirmed in the occurrence of various types of cancer^52–57^. Studies have shown that in some cases, the increase in promoter methylation levels is directly correlated with gene expression^33^. Compared with normal tissues, the promoter methylation level of SLC35A3 in CRC is significantly decreased. These results suggest that SLC35A3 may affect the progression of CRC through mutations or regulation of promoter methylation. However, these speculations still need to be further explored in future studies.

To investigate the role of SLC35A3 in CRC, we performed GO, KEGG, and GSEA analyses on differentially expressed genes between the high SLC35A3 group and the low SLC35A group based on the TCGA-COADREAD dataset. The GO analysis results showed that SLC35A3 and its differentially expressed genes may be involved in changes in cell membrane potential, transmembrane transport protein activity, and intercellular communication. The KEGG analysis results showed that the most significant enriched pathway was neuroactive ligand-receptor interaction. Neuroactive ligand-receptor interaction is known to be involved in the occurrence and development of breast cancer^58^, hepatocellular carcinoma^59^, renal cell carcinoma^60^, glioma^61^, and other cancer types. It can be seen that SLC35A3 and its differentially expressed genes may be involved in cell signaling transduction and the "neuroactive ligand-receptor interaction" pathway, regulating the occurrence and development of CRC. In the GSEA analysis, several pathways corresponding to the overexpression phenotype of SLC35A3 were highly enriched, including starch and sucrose metabolism, cell cycle, DNA double-strand break repair, base excision repair, epigenetic regulation of gene expression, and histone arginine methylation. These pathways are closely related to energy metabolism of CRC, cell proliferation, DNA damage repair, maintenance of genomic stability, and epigenetic regulation of gene expression^62–65^. Additionally, in the low-expression phenotype of SLC35A3, WNT signaling pathway, cancer pathways, and various invasive markers of cancer were significantly enriched, suggesting that SLC35A3 may play a role in regulating cancer signaling pathways, the occurrence and invasion of CRC. To validate the relationship between SLC35A3 expression and the phenotypes of CRC cells, we constructed an overexpression vector for SLC35A3. The effects of SLC35A3 overexpression on the proliferation, invasion, and apoptosis of CRC cells (HCT116 and SW620) were analyzed by CCK8, Transwell, and flow cytometry assays. The results showed that SLC35A3 overexpression significantly inhibited the proliferation and invasion of HCT116 and SW620 cells. Flow cytometry analysis further confirmed that SLC35A3 can significantly promote apoptosis of CRC cells, indicating that SLC35A3 can inhibit the proliferation of CRC cells by inducing apoptosis.

The immune microenvironment can affect the progression and efficacy of CRC and is closely related to clinical prognosis^34,66^. Understanding the tumor microenvironment, including immune cell infiltration, helps to reveal the mechanisms of CRC occurrence and development. Therefore, in order to describe the level of immune infiltration in CRC, we evaluated the potential impact of SLC35A3 on immune cell infiltration in the tumor microenvironment and the potential immune mechanisms mediated by SLC35A3. The results showed that SLC35A3 was widely associated with immune infiltration, including T-helper cells, Th2 cells, central memory T cells (Tcm cells), natural killer (NK) cells, plasmacytoid dendritic cells (pDCs), and regulatory T cells (Treg cells), which play key roles in the regulation of CRC prognosis. Among them, SLC35A3 was positively correlated with the infiltration levels of T-helper cells, Tcm cells, and Th2 cells, and negatively correlated with NK cells, Treg cells, and pDC cells. These correlations suggest that SLC35A3 can recruit T-helper cells, Tcm cells, and Th2 cells to the tumor microenvironment and prevent the recruitment of Treg cells, which promote immune tolerance and angiogenesis. CD4 is a transmembrane glycoprotein expressed on the surface of Th cells and plays an important role in immune response. CD4 + Th cells expressing CD4 coordinate immune responses by acting as effector cells or memory cells^67^. Among the above immune cells, infiltration of helper T cells showed the highest correlation with SLC35A3 expression. Therefore, we verified the correlation between SLC35A3 and infiltration of helper T cells through immunohistochemistry. The results showed that SLC35A3 expression was positively correlated with the expression of CD4, which is consistent with the above analysis results. In addition, we also investigated the correlation between SLC35A3 expression and immune checkpoints, and found that SLC35A3 expression was correlated with the expression of most immune checkpoints. Among them, SLC35A3 was positively correlated with the expression of PD-L1, ICOS, TIGIT, and CD40LG, and negatively correlated with the expression of PD-1, CD40, LAG3, and CD70. These results suggest that SLC35A3 may not only serve as a prognostic biomarker for CRC, but also as a potential target for immunotherapy, affecting the tumor immune microenvironment of CRC.

To our knowledge, there are currently no reports on the correlation between SLC35A3 and the prognosis of CRC. The importance and originality of this study lie in its systematic investigation of the relationship between SLC35A3 and CRC. However, this study still has some limitations: (1) There is no experimental verification of the impact of SLC35A3 methylation status on the phenotypes of CRC cells; (2) The effects of SLC35A3 on the malignant behavior of CRC cells were only explored in vitro experiments, and further evidence from in vivo experiments needs to be obtained in future studies; (3) The correlation between SLC35A3 expression and immune efficacy in CRC patients still needs to be further explored. In the future, we will fill these deficiencies and refine relevant experiments. However, overall, we have proposed for the first time the value of SLC35A3 in the prognosis and immune regulation of CRC patients. These findings help to improve the molecular profile of CRC and may provide new insights for the treatment of CRC.

## Conclusion

We have conducted a comprehensive bioinformatics analysis to investigate the role of SLC35A3 in colorectal cancer (CRC), including its expression, clinical prognosis, diagnosis, genetic changes, promoter methylation, metabolic regulation, and immune cell infiltration. Furthermore, our experimental validation confirmed the high expression of SLC35A3 in CRC tissue, which was positively correlated with the infiltration of helper T cells, suggesting its favorable impact on anti-tumor immune response in CRC patients. Cell experiments demonstrated that elevated expression of SLC35A3 can inhibit the proliferation and invasion of colon cancer cells while promoting apoptosis. In conclusion, SLC35A3 is a novel potential biomarker associated with prognosis and immune infiltration in CRC patients. This study lays the foundation for further exploration of the mechanism of SLC35A3 in CRC occurrence and treatment. However, further research is needed to verify our findings (Supplementary Information).

### Supplementary Information

Supplementary Figures.

## Data Availability

The data provided in this study can be obtained from the article materials.
