# Supplementary figures and images for: Colorectal cancer with low SLC35A3 is associated with immune infiltrates and poor prognosis
Source: Sci Rep. 2024 Jan 3;14:329. doi: 10.1038/s41598-023-51028-w (PMC10764849; doi:10.1038/s41598-023-51028-w)

**Unprocessed WB original images**

**HCT116（β-Actin）**

**
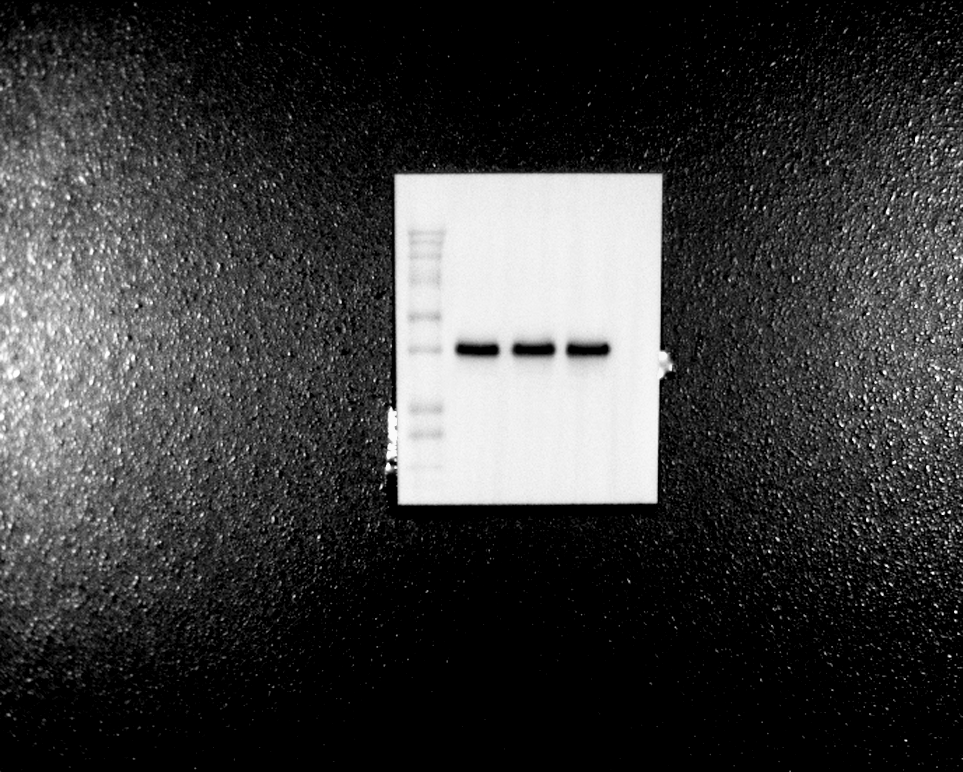
**

**HCT116（SLC35A3）**

**
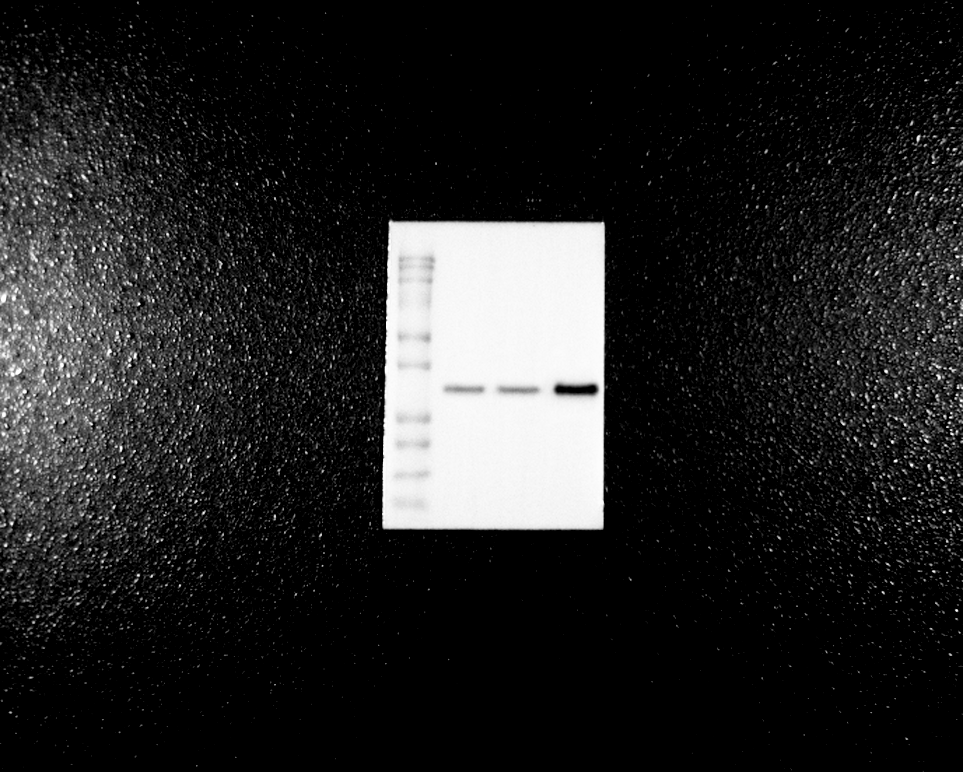
**

**SW620（β-Actin）**

**
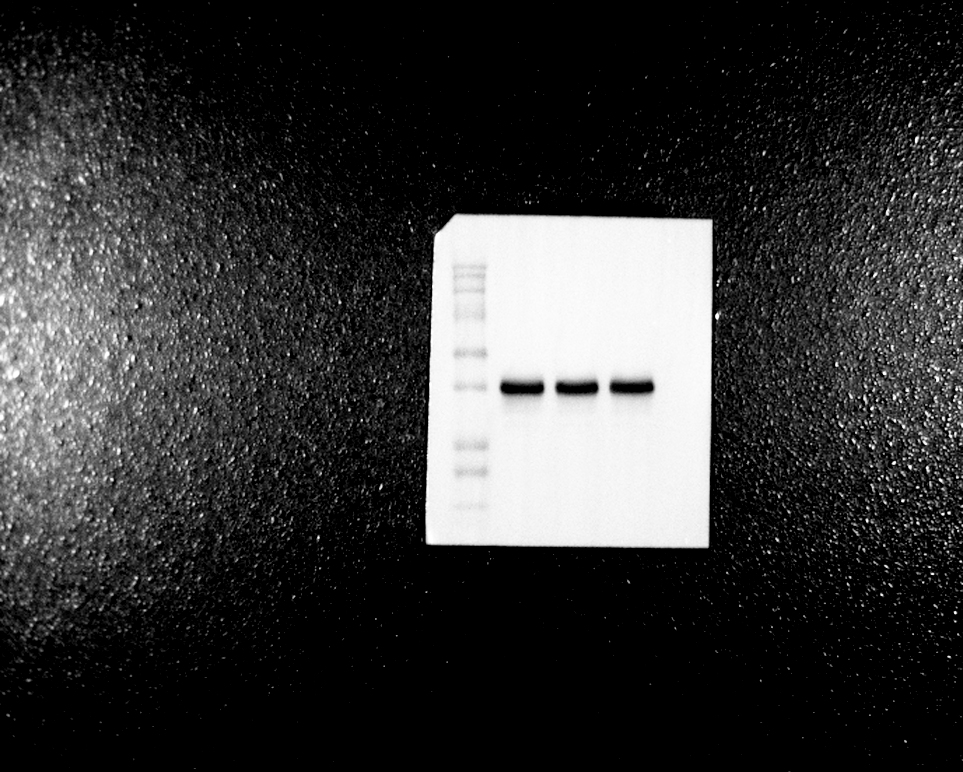
**

**SW620（SLC35A3）**

**
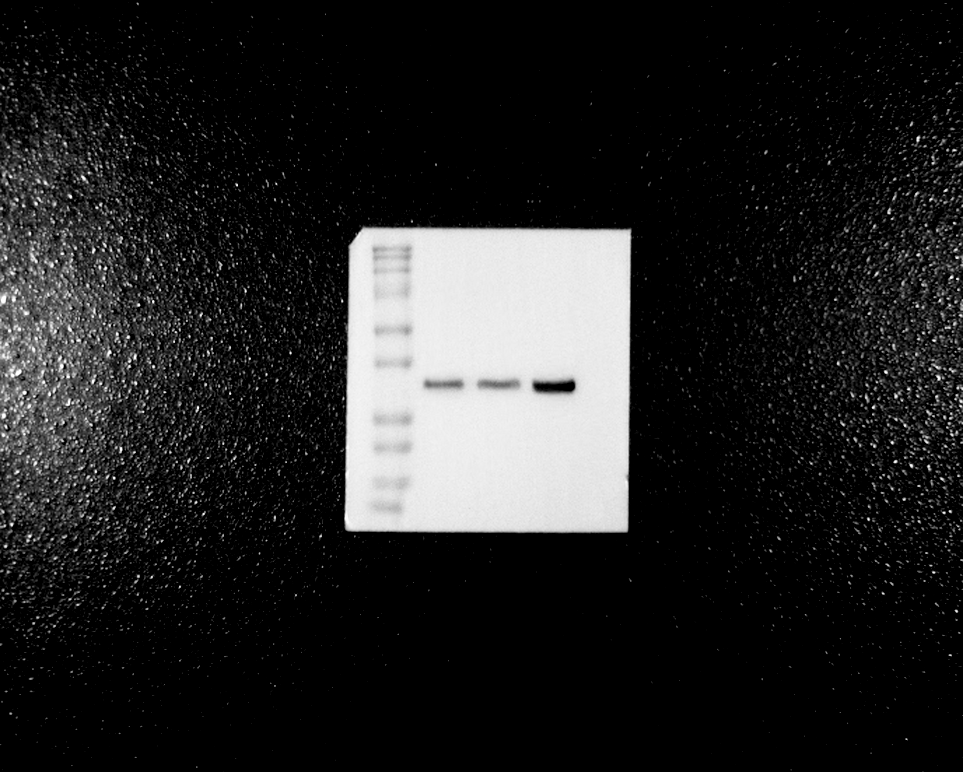
**

Supplement: Supplementary file 1 — Supplementary Figures. [file 41598_2023_51028_MOESM1_ESM.docx]
